# Universal framework for edge controllability of complex networks

**DOI:** 10.1038/s41598-017-04463-5

**Published:** 2017-06-26

**Authors:** Shao-Peng Pang, Wen-Xu Wang, Fei Hao, Ying-Cheng Lai

**Affiliations:** 1 The Seventh Research Division, School of Automation Science and Electrical Engineering, Beihang University, Beijing, 100191 China; 2 Science and Technology on Aircraft Control Laboratory, Beihang University, Beijing, 100191 China; 3 School of Systems Science, Beijing Normal University, Beijing, 100875 P. R. China; 4 School of Electrical, Computer and Energy Engineering, Arizona State University, Tempe, Arizona 85287 USA; 5 Department of Physics, Arizona State University, Tempe, Arizona 85287 USA

## Abstract

Dynamical processes occurring on the edges in complex networks are relevant to a variety of real-world situations. Despite recent advances, a framework for edge controllability is still required for complex networks of arbitrary structure and interaction strength. Generalizing a previously introduced class of processes for edge dynamics, the switchboard dynamics, and exploit- ing the exact controllability theory, we develop a universal framework in which the controllability of any node is exclusively determined by its local weighted structure. This framework enables us to identify a unique set of critical nodes for control, to derive analytic formulas and articulate efficient algorithms to determine the exact upper and lower controllability bounds, and to evaluate strongly structural controllability of any given network. Applying our framework to a large number of model and real-world networks, we find that the interaction strength plays a more significant role in edge controllability than the network structure does, due to a vast range between the bounds determined mainly by the interaction strength. Moreover, transcriptional regulatory networks and electronic circuits are much more strongly structurally controllable (SSC) than other types of real-world networks, directed networks are more SSC than undirected networks, and sparse networks are typically more SSC than dense networks.

## Introduction

Complex networks composed of interacting dynamical units are widespread in many natural, social and technological systems^1–5^. Great deal of effort has been devoted in the past decade to understand the evolution of complex networks and the interplay between network structures and dynamical processes^6, 7^. However, the problem of controlling complex networks^8–14^ remains unresolved as it is challenging to apply the classical control theory^15–17^ to complex networks. Liu *et al*. made a breakthrough by developing a structural controllability theory^18, 19^ for complex networks, and offering a mathematical foundation and efficient computational algorithms based on the concept of maximum matching to characterize the controllability of directed networks^20^. An exact controllability theory was then developed to characterize and analyze the controllability of undirected networks^21^. The key issue underpinning these works on network controllability is to identify a minimum set of driver nodes in a network to steer the network system to any desired final state within finite time^15–17^. Due to the importance of the network control problem, recent years have witnessed a growing interest in investigating various aspects of controllability of complex networks^22–37^.

Most studies of network controllability focused on nodal dynamical processes, in which the variables are defined on individual nodes and the interactions occur exclusively among the neighboring nodes. However, in many real-world networks, edge dynamics can also be important. For example, in the Internet with computers and routers (a directed network), the edges represent physical connections such as Ethernet cables, optical fiber cables, and wireless connections, enabling nodes to transmit information. A node (e.g., a router) processes the information received from its inbound edges and decides to which nodes the information is transmitted through some outbound edges. The state variables are the inbound and outbound signals, and their dynamical evolutions are governed by the switching matrices. The state variables, together with the switching matrices, define the edge dynamics. Another example is railway networks, which are undirected, where the nodes represent stations and two nodes are connected by an edge if there is at least one train that stops at both nodes. A node receives and sends trains through the corresponding edge connected with it. The state variables are the inbound and outbound trains on an edge, and the transportation rule is modeled by the switching matrices. A pioneering work to address the edge controllability of complex networks was proposed by Nepusz and Vicsek^38^. They introduced the switchboard dynamics as a general mathematical framework for edge dynamics and discovered that the structural controllability of edge dynamics can differ characteristically from that of nodal dynamics. Specifically, they considered directed complex networks *G*(*V*, *E*) with **x** denoting the vector specifying the state of each edge in the network, and with ${{\bf{y}}}_{v}^{-}$ and ${{\bf{y}}}_{v}^{+}$ being the state vectors corresponding to the incoming and outgoing edges of node *v*, respectively. Factors that can influence the evolution of the state vector ${{\bf{y}}}_{v}^{+}$ are the vector ${{\bf{y}}}_{v}^{-}$, the vector of the damping terms ***τ***_*v*_, and the external input vector **u**_*v*_. The edge dynamical process can then be described by the following switchboard dynamics:

1 $${\dot{{\bf{y}}}}_{v}^{+}={S}_{v}\cdot {{\bf{y}}}_{v}^{-}-{{\boldsymbol{\tau }}}_{v}\otimes {{\bf{y}}}_{v}^{+}+{\sigma }_{v}{{\bf{u}}}_{v},$$

where ${S}_{v}\in {{\mathbb{R}}}^{{k}_{v}^{+}\times {k}_{v}^{-}}$ is *switching matrix*. Its row number equals the out-degree ${k}_{v}^{+}$ and its column number is the in-degree ${k}_{v}^{-}$ of node *v*. *σ*_*v*_ is unity if node *v* is a driver node and is zero otherwise. ⊗ denotes the entry-wise product of the two vectors of the same size. This switchboard dynamics is suitable for modeling a variety of real world situations such as social communications and load-balancing or routing on the Internet^38, 39^.

**Figure 1:**
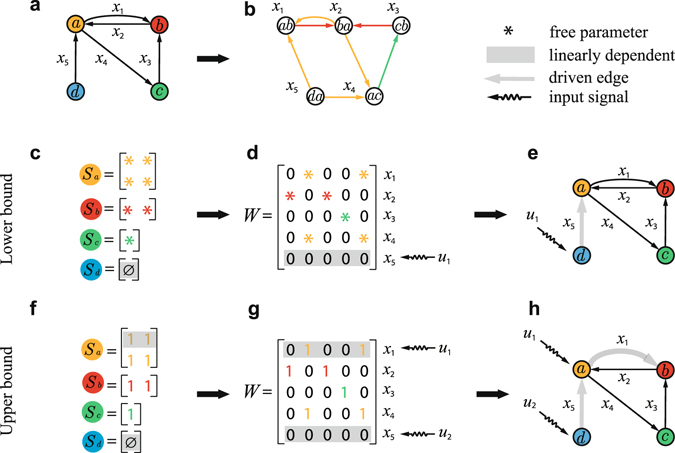
Control of general switchboard dynamics. (**a**) A directed network *G* with four nodes: *a*, *b*, *c*, and *d*, and five edges: *x*_*i*_ (*i* = 1, …, 5). (**b**) The line graph *L*(*G*) of the original directed network *G*. The colors of edges in *L*(*G*) correspond to those of the nodes in (**a**). (**c**) Structural switching matrices of the nodes in network *G* in (**a**). (**d**) Structural adjacency matrix *W* of the line graph *L*(*G*) in (**b**). (**e**) Driver node, driven edge and input signal for the structural adjacency matrix *W*. (**f**) Unweighted switching matrices of the nodes in network *G* in (**a**). (**g**) Unweighted adjacency matrix *W* of the line graph *L*(*G*) in (**b**). (**h**) Driver node, driven edge and input signals for the structural adjacency matrix *W*. Panels (**c**–**e**) and (**f**–**h**) correspond to the lower and upper bounds of controllability, respectively. The linearly dependent rows in *W* in (**d**,**g**) stem from independent rows in the switching matrices in (**c**,**f**), respectively. The edges associated with linearly dependent rows in *W* are the driven edges that should be controlled. The external input signals *u* should be imposed on the tail nodes of the driven edges.

Reformulating the switchboard dynamics in terms of the edge variables yields a time invariant dynamical system:

2 $$\dot{{\bf{x}}}=(W-T)\cdot {\bf{x}}+H\cdot {\bf{u}},$$

where $W\in {{\mathbb{R}}}^{M\times M}$ is the transpose of the adjacency matrix of the line graph *L*(*G*) (see Fig. 1(a,b) for an example of a line graph), in which *w*_*ij*_ is nonzero if and only if the head of edge *j* is the tail of edge *i*. *T* is a diagonal matrix composed of the damping terms of each edge. *H* is a diagonal matrix where the *i* th element is *σ*_*v*_ if node *v* is the tail of edge *i*. The controllability of system (2) can be assessed and quantified by employing the structural control theory through the assumption that *W* − *T* is a structural matrix, while omitting the effect of interaction strengths in the switching matrix^18, 20^. Consequently, a minimum set of driven edges in the original network *G* can be identified by calculating the maximum matching of the line graph *L*(*G*). All the tail nodes of the driven edges are the driver nodes in *G*. One key result is that all divergent nodes (${k}_{v}^{+} > {k}_{v}^{-}$) are driver nodes, and that one arbitrary node from each balanced component (${k}_{v}^{+}={k}_{v}^{-} > 0$ for all nodes in a connected component) is also a driver node^38^. The criterion for discerning driver nodes gives rise to several structural controllability properties of edge dynamics that differ markedly from those associated with nodal dynamics. Other findings include that most of real-world networks are more controllable than their randomized counterparts, transcriptional regulatory networks are easy to be controlled, heterogeneous networks are more controllable than homogeneous networks, and a positive correlation between the in- and out-degrees can enhance the controllability^38^. Despite the interesting findings, since the structural controllability theory is valid only for directed networks, a number of open issues remain, such as the edge controllability of undirected networks, the effect of interaction strengths on the controllability, and the strong structural controllability associated with edge dynamics. It is worth of noting that strong structural controllability is an important index for measuring the robustness of controllability against uncertainties or variations in the interaction strengths among the edges.

In this paper, we generalize the existing framework of structural edge controllability^38^ by developing a universal framework capable of characterizing the controllability of edge dynamics in *arbitrary* networks and interaction strengths. In particular, by bridging the exact controllability theory for nodal dynamics and the general switchboard dynamics, we find that, for an arbitrary network with any distributions of the interaction strengths among the edges, the role of a node in edge dynamics and strong structural controllability is exclusively determined by the local weighted structure of the node. We use this key result to uncover a number of phenomena associated uniquely with edge controllability, which have no counterparts in nodal controllability. Firstly, the set of driver nodes in edge controllability is unique in an arbitrary network, whereas for nodal controllability, there are many configurations of driver node sets in spite of the fixed number of driver nodes. The set of strongly structurally controllable (SSC) nodes is unique and is fully determined by the local topology of each node, whereas for nodal controllability, there exists no criterion to identify the SSC nodes. Secondly, interaction strengths among the edges play a more significant role in edge controllability than the network structure does. Particularly, there exist lower and upper bounds of edge controllability, which are determined by the interaction strengths. We prove rigorously that the lower and upper bounds are determined by the structural switching and the unweighted switching matrices, respectively. In fact, there is a vast range between the bounds, in which a broad spectrum of controllability can be achieved. Thirdly, applications of our framework to real-world networks show that transcriptional regulatory networks and electronic circuits have higher strong structural controllability. In addition, real directed networks are more SSC than undirected networks, and sparser networks are more SSC than denser networks. For all the results concerning general edge controllability and strongly structural controllability, we provide analytic formula and results from extensive numerical tests. We emphasize the universal nature of our edge controllability framework: it is applicable to arbitrary network structure (e.g., directed or undirected, weighted or unweighted) and interaction strengths among the edges. In fact, we demonstrate that for directed networks with structural switching matrices, a number of key results reduce to those of structural edge controllability.

## Results

### General switchboard dynamics

In the original switchboard dynamics^38^, the switching matrix *S*_*v*_ must be a structural matrix, in which all nonzero elements are independent free parameters. Instead, we release the restriction of structural matrix *S*_*v*_ and consider a general switchboard dynamics with any kind of switching matrices, in which the elements capture the interaction strengths among the edges. We exemplify two typical switching matrices, the weighted switching matrix and the unweighted switching matrix. In the former, all nonzero elements can be any values, and in the latter, all nonzero elements are one.

For directed networks, the general switchboard dynamics (GSBD) is described by Equation (2). The adjacency matrix *W* of the line graph is of the same type as *S*_*v*_ of each node (Fig. 1). In contrast, for undirected networks, Equation (2) cannot be immediately adopted because, associated with each edge, the interaction and transmission are bidirectional. Two neighboring edges connecting with the same node can be the input and output of each other. To define GSBD for undirected networks, we split each undirected edge into two directed edges of opposite directions and use a pair of state variables, $({x}_{i},{x}_{i}^{^{\prime} })$, to denote such an edge, where each variable corresponds to one of the directed edges. The state vector of the dynamical process occurring on undirected edges is then ${\bf{x}}={({x}_{1},{x}_{1}^{^{\prime} },\ldots ,{x}_{M},{x}_{M}^{^{\prime} })}^{T}$. The switching matrix can be written as ${S}_{v}\in {{\mathbb{R}}}^{{k}_{v}\times {k}_{v}}$, where *k*_*v*_ is the degree of node *v*. For the whole network, the dynamical process can still be described by Equation (2), but the dimension of the matrix *W* is doubled: $W\in {{\mathbb{R}}}^{2M\times 2M}$, where *M* is the number of undirected edges in *G*. The properties of *W* are still determined by *S*_*v*_ (see Supplementary Fig. S1 for an illustration). Our GSBD thus provides a more general characterization of the dynamics occurring on edges for arbitrary networks. The focus of our study is on the effect of the interaction strengths in the switching matrix on edge controllability. For a general switching matrix *S*_*v*_, the conventional structural control theory^20^ is not applicable, due to the non-uniform interaction strengths among the edges and the undirected nature of the network structure. This calls for a more general control theory to determine/quantify the edge controllability.

### Controllability framework of the GSBD

We make use of the exact controllability theory^21^ developed recently to determine the controllability of the GSBD. By definition, the controllability of a network is measured by the minimum number *N*_D_ of driver nodes. Prior to the identification of the driver nodes, we must ascertain the minimum number *M*_D_ of the driven edges as determined by the matrix *W* − *T* in Equation (2). According to the exact controllability theory^35^, the damping matrix *T* with identical diagonal elements has no effect on the controllability of the network characterized by *W* − *T*, which can be proven rigorously. That is, the set of driven edges and driver nodes will not be affected by *T*, so it can be neglected. As a result, all self-loops of nodes stemming from −*T* in the line graph *L*(*G*) can be eliminated (see Fig. 1). We then determine *M*_D_ of *G* characterized by the adjacency matrix *W* and *N*_D_ of *L*(*G*), and present our key results.

Figure 1 shows two representative cases of a simple directed network with structural and unweighted switching matrices. We can prove that the former and the latter cases generate the lower and upper bounds, respectively, of both *M*_D_ and *N*_D_ for any network (see Methods). The line graph *L*(*G*) is shown in Fig. 1(b). The driven edges in *G* correspond to the driver nodes in *L*(*G*). According to the exact controllability theory and the properties of line graphs, we can prove that the minimum number of driver nodes in *L*(*G*) (driven edges in *G*) is *M* − rank(*W*), where *M* is the number of edges in *G* (see Supplementary Note 1). That is, *M*_D_ is the number of linearly dependent rows in *W*. For example, in the structural matrix *W* of Fig. 1(d), the row corresponding to *x*_5_ is linearly dependent on the other rows, indicating that a control signal should be applied to *x*_5_. Figure 1(g) illustrates linearly dependent rows in the unweighted matrix *W*. Making use of a generic feature (see Methods and Supplementary Note 2) of line graphs, we obtain our first key result: the linearly dependent rows in *W* are exclusively determined by the linearly dependent rows in the switching matrices *S*_*i*_ of all nodes. As shown in Fig. 1, the linearly dependent row (*x*_5_) in *W* (Fig. 1(d)) stems from *S*_*d*_ (Fig. 1(c)) with a null set. The other rows in *W* are linearly independent because *S*_*a*_, *S*_*b*_ and *S*_*c*_ in Fig. 1(c) are row-full rank. Similarly, for the unweighted *W* in Fig. 1(g), the two linearly dependent rows *x*_1_ and *x*_5_ originate from *S*_*a*_ and *S*_*d*_ in Fig. 1(f), respectively. This key finding indicates that driven edges can be identified from the switching matrices *S*_*i*_ by using the local information of nodes without relying on the line graph *L*(*G*), which gives

3 $${M}_{{\rm{D}}}=M-\sum _{i=1}^{N}{\rm{rank}}({S}_{i})+\sum _{i=1}^{C}{\beta }_{i},$$

where *C* is the number of connected components in *G*. *β*_*i*_ = 1 if the switching matrices of all nodes in component *i* are square matrices with full rank, and *β*_*i*_ = 0 otherwise. The second term from each connected component has little effect on *M*_D_. As a result, *M*_D_ is determined by the rank of *S*_*i*_.

After the driven edges are determined, we can immediately specify the driver nodes at the tail end of the driven edges, ensuring that external control signals applied to the driver nodes can directly pass on to the driven edges. As shown in Fig. 1(e,h), the driver nodes at the tail end of the driven edges can be identified based on local information contained in the switching matrices in Fig. 1(c,f), which does not depend on the global structure of *L*(*G*). In general, according to the characteristics of the driven edges and the relation between the driven edges and the driver nodes, we can prove that *S*_*i*_ associated with driver node *i* satisfies

4 $${\rm{rank}}({S}_{i}) < {k}_{i}^{+},$$

where ${k}_{i}^{+}$ is the out-degree of *i*. This means that, if the switching matrix of a node is not full row-rank, the node must be a driver node. This is the general criterion for identifying the driver nodes based only on the local information of each node. From this result, the number *N*_D_ of the driver nodes can be calculated through

5 $${N}_{{\rm{D}}}={N}_{({\rm{rank}}({S}_{i}) < {k}_{i}^{+})}+\sum _{i=1}^{C}{\beta }_{i},$$

where *β*_*i*_ is the same as that in Equation (3). Analogous to the expression of *M*_D_, the second term in Equation (5) has little effect on *N*_D_. We refer to full row-rank as full rank in the remaining paper for simplicity.

It is worth noting that our edge controllability theory is not a trivial application of the exact controllability theory to the line graphs of a network. In particular, by exploiting the unique properties of a line graph, we prove that the controllability of a node is determined only by its local weight structure. As a result, the set of driver nodes in a general network with an arbitrary distribution of interaction strengths among the edges is unique. This is our key result here. Moreover, we also prove that, for the upper and lower controllability bounds, the driver nodes are determined only by the local topology of each node, enabling readily implementable algorithms to find the driver nodes associated with the bounds. As presented below, this key result can also address the issue of strong structural controllability, which is important for understanding the robustness of controlling networks in the presence of uncertainties or variations of interaction strengths among the edges.

### Universal controllability bounds

We can prove that there exist universal upper and lower bounds for edge controllability for any network, and that any value of the controllability in between can be achieved by adjusting the interaction strengths among the edges. In particular, the upper and lower bounds are reached if *S*_*i*_ of each node is an unweighted matrix and a structural matrix, respectively (see Methods for a detailed proof).

**Figure 2:**
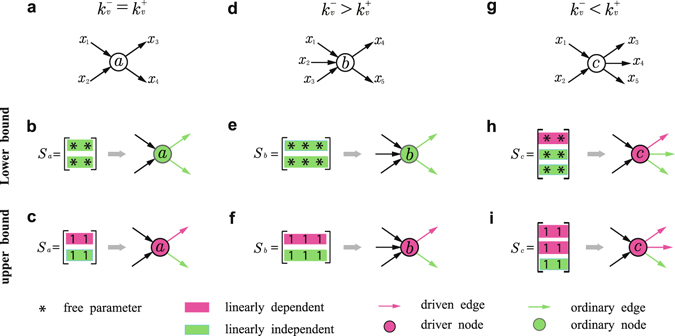
Classification of edges and nodes based on local information. (**a**) Node *a* with two incoming and outgoing edges. (**b**) Structural switching matrix *S*_*a*_ of node *a* and the category of node *a* and its outgoing edges. *S*_*a*_ is row-full rank, so the two outgoing edges are non-essential (ordinary) edges and *a* is a non-essential node. (**c**) Unweighted switching matrix *S*_*a*_ and the category of node *a* and its outgoing edges. In *S*_*a*_, there is a linearly dependent row corresponding to a driven outgoing edge. Node *a* at the tail end of the driven edge is a driver node. (**d**) Node *b* with three incoming edges and two outgoing edges. (**e**) Structural switching matrix *S*_*b*_ with row-full rank and the category of node *b* and its outgoing edges. Node *b* and its outgoing edges are non-essential. (**f**) Unweighted switching matrix *S*_*b*_ and the category of node *b* and its outgoing edges. The row-rank of *S*_*b*_ is unity, leading to one driven edge. Node *b* becomes a driver node with one driven edge. (**g**) Node *c* with two incoming edges and three outgoing edges. (**h**) Structural switching matrix *S*_*c*_ with deficient row-rank and the category of node *b* and its outgoing edges. The row-rank of *S*_*c*_ is 2, so there are two non-essential edges. Node *c* is a driver node with one driven edge. (**i**) Unweighted switching matrix *S*_*c*_ and the category of node *c* and its outgoing edges. The row-rank of *S*_*c*_ is unity, indicating one non-essential edge and two driven edges. Node *c* with two driven outgoing edges is thus a driver node. The structural switching matrices in (**b**), (**e**,**h**) correspond to the lower bound, and the unweighted switching matrices in (**c**), (**f**,**i**) are associated with the upper bound.

In general, it is necessary to calculate the rank(*S*_*i*_) of all nodes to identify the driver nodes and to obtain *N*_D_ and *M*_D_. However, for structural or unweighted *S*_*i*_, we are able to identify the driver nodes and the driven edges based solely on their in-degrees ${k}_{v}^{-}$ and out-degrees ${k}_{v}^{+}$ without having to calculate rank(*S*_*i*_). As shown in Fig. 2, nodes in terms of their in- and out-degrees can be classified into three categories: (I) ${k}_{v}^{-}={k}_{v}^{+}$, (II) ${k}_{v}^{-} > {k}_{v}^{+}$, and (III) ${k}_{v}^{-} < {k}_{v}^{+}$. For case (I), e.g., node *a* in Fig. 2(a), the structural *S*_*a*_ is always full rank (Fig. 2(b)), indicating that node *a* is *non-essential* in the sense that no external input signal is needed to control the outgoing edges of *a*, and that all the outgoing edges of *a* are non-essential as well. In contrast, for the unweighted *S*_*a*_, rank(*S*_*a*_) is always unity and it satisfies the inequality ${\rm{rank}}({S}_{a}) < {k}_{a}^{+}$, as shown in Fig. 2(c). Thus, without any external input signal applied to the node, only one outgoing edge can be fully controlled, regardless of the degree of *a*. As a result, *a* in this case must be a driver node. Moreover, its one outgoing edge that can be arbitrarily selected is a non-essential edge, while the other outgoing edges are driven edges. For case (II), e.g., node *b* in Fig. 2(d), the structural *S*_*b*_ is always full rank (${\rm{rank}}({S}_{b})={k}_{b}^{+}$), indicating that node *b* and all of *b*’s outgoing edges are non-essential. This is similar to the structural matrix *S*_*a*_ in case (I), as shown in Fig. 2(e). In contrast, for the unweighted *S*_*b*_, despite the inequality ${k}_{b}^{-} > {k}_{b}^{+}$, the rank of *S*_*b*_ is always unity and *b* is a driver node with a single non-essential edge among all *b*’s outgoing edges, as shown in Fig. 2(f). This also implies that, for an unweighted switching matrix, the associated node must be a driver node if ${k}_{v}^{+} > 1$. For case (III), e.g., node *c* in Fig. 2(g), both the structural *S*_*c*_ (Fig. 2(h)) and the unweighted *S*_*c*_ (Fig. 2(i)) satisfy the inequality ${\rm{rank}}({S}_{c}) < {k}_{c}^{+}$. As a result, node *c* is a driver node for either structural or unweighted switching matrix, with different driven edges for the two scenarios.

From the above illustrations and arguments, we obtain our key result of identifying the driver nodes based on their ${k}_{v}^{+}$ and ${k}_{v}^{-}$ for the lower (structural switching matrix) and the upper (unweighted switching matrix) bounds. In particular, for a directed network, at the lower bound, a node with more outgoing than incoming edges must be a driver node. For the upper bound, a node with more than one outgoing edge must be a driver node, and a node without incoming edges and with a single outgoing edge must also be a driver node. For an undirected network, at the lower bound, a single driver node is required for each connected component, while for the upper bound, a node with more than one edge must be a driver node.

According to the criterion, for identifying the driver nodes and the driven edges, *N*_D_ and *M*_D_ associated with the lower and upper bounds can be calculated, as summarized in Table 1, where the contributions from isolated components are included (see Methods and Supplementary Note 3). Note that, for the lower bound in directed networks, our results reduce to those of structural edge controllability (see Supplementary Note 4). For the other scenarios, the results have not been reported prior to our work to our knowledge.

**Table 1:** Numbers of driver nodes and driven edges associated with upper and lower bounds.

	Directed	Undirected
${N}_{{\rm{D}}}^{{\rm{U}}}$	${N}_{({k}_{v}^{+} > 1)}+{N}_{({k}_{v}^{-}=\mathrm{0,}{k}_{v}^{+}=1)}+{\sum }_{i=1}^{C}{\beta }_{i}^{^{\prime} }$	${N}_{({k}_{v} > 1)}+{\sum }_{i=1}^{C}{\beta }_{i}^{^{\prime} }$
${N}_{{\rm{D}}}^{{\rm{L}}}$	${N}_{({k}_{v}^{-} < {k}_{v}^{+})}+{\sum }_{i=1}^{C}{\beta }_{i}^{^{\prime\prime} }$	${\sum }_{i=1}^{C}{\beta }_{i}^{^{\prime\prime} }$
${M}_{{\rm{D}}}^{{\rm{U}}}$	$M-{N}_{({k}_{v}^{-} > 0,{k}_{v}^{+} > 0)}+{\sum }_{i=1}^{C}{\beta }_{i}^{^{\prime} }$	$M-{N}_{({k}_{v} > 0)}+{\sum }_{i=1}^{C}{\beta }_{i}^{^{\prime} }$
${M}_{{\rm{D}}}^{{\rm{L}}}$	$M-{\sum }_{i=1}^{N}\,{\rm{\min }}\,\{{k}_{i}^{-},{k}_{i}^{+}\}+{\sum }_{i=1}^{C}{\beta }_{i}^{^{\prime\prime} }$	${\sum }_{i=1}^{C}{\beta }_{i}^{^{\prime\prime} }$

For general networks (directed or undirected), ${N}_{{\rm{D}}}^{{\rm{U}}}$ and ${M}_{{\rm{D}}}^{{\rm{U}}}$ are the numbers of driver nodes and driven edges associated with the upper bound, respectively. Similar notations hold for ${N}_{{\rm{D}}}^{{\rm{L}}}$ and ${M}_{{\rm{D}}}^{{\rm{L}}}$. The upper and lower bounds are associated with unweighted and structural switching matrices, respectively. *N* and *M* are the number of nodes and the number of edges in network *G*, respectively, and ${k}_{v}^{+}$ and ${k}_{v}^{-}$ are the out- and in-degree, respectively. The quantity ${\beta }_{i}^{^{\prime} }$ is unity if the *i* th connected component only contains nodes with ${k}_{v}^{+}={k}_{v}^{-}=1$; ${\beta }_{i}^{^{\prime} }=0$ otherwise. The quantity ${\beta }_{i}^{^{\prime\prime} }$ is unity if the *i* th connected component is balanced (${k}_{v}^{+}={k}_{v}^{-} > 0$ for all nodes); ${\beta }_{i}^{^{\prime\prime} }=0$ otherwise.

**Figure 3:**
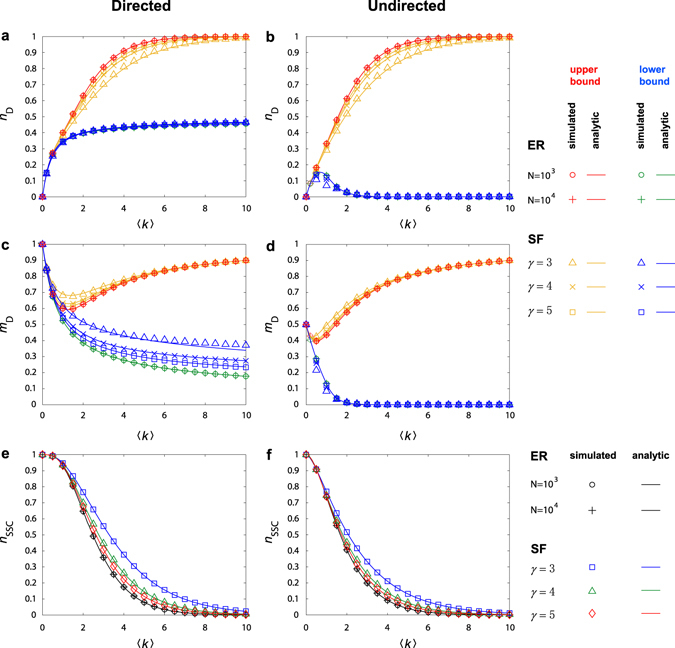
Controllability bounds and strong structural controllability of model networks. For directed and undirected ER and SF networks, (**a**,**b**) upper and lower bounds of *n*_D_, (**c**,**d**) upper and lower bounds of *m*_D_, and (**e**,**f**) strong structural controllability measure *n*_ssc_ as a function of the average degree 〈*k*〉. *γ* is the scaling exponent of the SF network. The data points are numerical results and the curves represent analytical formulas. All the numerical results are obtained by averaging over 50 independent networks realizations. The other parameters are the same as in Table 1. See Methods and Supplementary Note 9 for network models.

Next we will verify the universal controllability bounds for both directed and undirected model networks and offer further analytical results. According to Liu *et al*.^20^, the nodal controllability *n*_D_ can be defined as the ratio of the minimum number of driver nodes to the total number of nodes, i.e., *n*_D_ = *N*_D_/*N*. The edge controllability *m*_D_ can be defined in a similar way as *m*_D_ = *M*_D_/*M*, where *M* is the total number of edges in *G*. As shown in Fig. 3, the upper and lower bounds of *n*_D_ and *m*_D_ hold for both directed and undirected networks, regardless of whether the degree distribution is homogeneous or heterogeneous. In fact, the degree distribution has little effect on *n*_D_ and *m*_D_. Especially for undirected networks, the values of *n*_D_ and *m*_D_ for different undirected networks have nearly overlapping upper and lower bounds. Another remarkable result is that, except for very small values of the average degree 〈*k*〉, the range or “distance” between the upper and lower bounds is appreciable. Any value of *n*_D_ and *m*_D_ in between the bounds is achievable by properly setting the element values in the switching matrices *S*_*i*_. These results demonstrate that interaction strengths among the edges in the switching matrices play a more important role in edge controllability than the network structure does, in sharp contrast to the situation of controlling nodal dynamics (or nodal controllability). These findings provide a deeper understanding of the controllability of edge dynamics.

We also find a non-monotonic behavior in the upper bound of *m*_D_ for both directed and undirected networks, and in the lower bound of *n*_D_ for undirected networks as well. Such a behavior results from the combining effect of the first and the second terms in Equations (3) and (5), where the second term represents the contribution from each isolated component. Notice that the non-monotonic phenomenon occurs in the regime of relatively small values of 〈*k*〉, for which there are a number of isolated components. As 〈*k*〉 increased, the contribution from the second term diminishes because of the reduction in the number of isolated components, whereas the contribution from the first term begins to dominate, providing an explanation for the non-monotonic behavior.

We derive the analytical results of the bounds for different networks according to the classification of nodes and edges from local information, as shown in Fig. 2. The results are presented in Table 2 where, except for *m*_D_ of directed networks associated with the lower bound, closed-form formulas can be obtained (see Supplementary Note 5 for detailed derivations). For directed networks, the analytical results of the lower bound reduce to the previous results of structural edge controllability, providing further validation of our theory.

**Table 2:** Analytical results of controllability for upper and lower bounds.

		Directed	Undirected
ER	${n}_{{\rm{D}}}^{{\rm{U}}}$	1 − (〈*k*〉 + 1)*e* ^−〈*k*〉^ + 〈*k*〉*e* ^−2〈*k*〉^	$1-(\langle k\rangle +1){e}^{-\langle k\rangle }+\frac{\langle k\rangle }{2}{e}^{-2\langle k\rangle }$
	${n}_{{\rm{D}}}^{{\rm{L}}}$	$\frac{1}{2}(1-{e}^{-2\langle k\rangle }{I}_{0}(2\langle k\rangle ))$	*n*_CC_ − *e* ^−〈*k*〉^
	${m}_{{\rm{D}}}^{{\rm{U}}}$	$1-\frac{1}{\langle k\rangle }(1-2{e}^{-\langle k\rangle }+{e}^{-2\langle k\rangle })$	$1-\frac{1}{\langle k\rangle }(1-{e}^{-\langle k\rangle })+\frac{1}{2}{e}^{-2\langle k\rangle }$
	${m}_{{\rm{D}}}^{{\rm{L}}}$	$\frac{{e}^{-2\langle k\rangle }}{\langle k\rangle }{\sum }_{j=1}^{\infty }j{I}_{j}(2\langle k\rangle )$	$\frac{1}{\langle k\rangle }({n}_{{\rm{CC}}}-{e}^{-\langle k\rangle })$
SF	${n}_{{\rm{D}}}^{{\rm{U}}}$	$1-\frac{1}{\zeta (\gamma )}$	$1-\frac{1}{\zeta (\gamma )}+\frac{1}{2\zeta (\gamma )\zeta (\gamma -1)}$
	${n}_{{\rm{D}}}^{{\rm{L}}}$	$\frac{1}{2}-\frac{\zeta (2\gamma )}{2\zeta {(2\gamma )}^{2}}$	*n*_CC_
	${m}_{{\rm{D}}}^{{\rm{U}}}$	$1-\frac{\zeta (\gamma )}{\zeta (\gamma -1)}$	$1-\frac{\zeta (\gamma )}{\zeta (\gamma -1)}+\frac{1}{2\zeta {(\gamma -1)}^{2}}$
	${m}_{{\rm{D}}}^{{\rm{L}}}$	$\frac{{\sum }_{j=1}^{\infty }j{\sum }_{i=1}^{\infty }{i}^{-\gamma }{(i+j)}^{-\gamma }}{\zeta (\gamma )\zeta (\gamma -1)}$	$\frac{1}{\langle k\rangle }{n}_{{\rm{CC}}}$

Formulas for ${n}_{{\rm{D}}}^{{\rm{U}}}$ and ${m}_{{\rm{D}}}^{{\rm{U}}}$ (upper bounds) and ${n}_{{\rm{D}}}^{{\rm{L}}}$ and ${m}_{{\rm{D}}}^{{\rm{L}}}$ (lower bounds) for directed and undirected ER and SF (*κ* → ∞) networks. The average degree is 〈*k*〉 = 〈*k* ^in^〉 = 〈*k* ^out^〉 = *M*/*N*, *I*_*a*_(*x*) is the modified Bessel function of the first kind, *ζ*(*x*) is the Riemann zeta function, and *n*_C*C*_ is the expected fraction of isolated components in the ER undirected networks. Note that the average degree is 〈*k*〉 = *ζ*(*γ*)/*ζ*(*γ* − 1) for SF networks with parameter *κ* → ∞ (see Supplementary Note 5 for details).

In addition to ER and SF networks, simulation and analytical results of model networks with an exponential degree distribution and with a power-law degree distribution are provided in Supplementary Note 5, Figs S2 and S3. The results are qualitatively the same as the results in the main text. The analytical results of some simple and regular networks are also provided in Supplementary Note 5 and Table S1. The transition between the upper and lower bounds has also been analyzed, as presented in Supplementary Note 6 and Fig. S4.

### Strong structural controllability

Strong structural controllability is a critical notion of quantification if the controllability of a network is robust against uncertainties or fluctuations in the interaction strengths. At present, to establish strong structural controllability even for nodal dynamics remains to be a challenging problem. Remarkably, we find that, for edge dynamics, strong structural controllability can be related to the controllability bounds in a straightforward manner, thus allowing us to obtain a straightforward but appealing analytic criterion to quantify strong structural controllability.

A network is SSC if, regardless of the values of the elements in the switching matrices, the quantities *n*_D_ and *m*_D_ do not change^20, 40^. This means that the controllability of a fully SSC network cannot be affected by variations in the interaction strengths among the edges, exclusively determined by the network structure. Because the category of a node is determined only by its local information (Fig. 2), we can determine if a node is SSC based only on its switching matrix. In particular, a node is SSC if the nodal and edge categories do not change for any values of the elements in its switching matrix. We can prove that a node with ${k}_{v}^{+}\le 1$ or ${k}_{v}^{-}\le 1$ is SSC for an arbitrarily directed network and a node with *k*_*v*_ ≤ 1 is SSC for an arbitrary undirected network (see Methods).

The strong structural controllability of a network can be defined as the ratio of the number *N*_SSC_ of the SSC nodes to the network size *N*, i.e.,

6 $${n}_{{\rm{ssc}}}=\frac{{N}_{{\rm{ssc}}}}{N}\mathrm{.}$$

A network with a higher value of *n*_ssc_ is more SSC, and a network with *n*_ssc_ = 1 is fully SSC. As shown in Fig. 3(e,f), sparse networks with small values of 〈*k*〉 are nearly fully SSC. This is also reflected in Fig. 3(a–d) in the regime of small 〈*k*〉, where the upper and lower bounds are equal. Observe that *n*_ssc_ decreases as 〈*k*〉 is increased, which means that sparser networks are generally more SSC than denser networks. The dependence of *n*_ssc_ on 〈*k*〉 can be analytically predicted (Supplementary Note 7).

Strong structural controllability is related with the controllability bounds, in the sense that the upper and lower bounds of a fully SSC network coincide with each other. This can be explained, as follows. The controllability bounds are determined by the interaction strengths. For a fully SSC network, its edge controllability is not affected by the interaction strengths. That is, interaction strengths do not induce any difference between the upper and lower bounds in an SSC network. As a result, the controllability bounds must be exactly the same in a fully SSC network.

### Controllability properties of real-world networks

Our theoretical framework and analytic predictions enable us to study the edge controllability of a variety of real directed and undirected networks. The upper and lower bounds of *n*_D_, *m*_D_ and *n*_ssc_ for different types of real networks are shown in Table 3. An interesting finding is that electronic circuits^41^ and regulatory networks are more SSC, including the ownership network of US telecommunications and media corporations^42^ and the transcriptional regulatory networks of Echerichia Coli^41^ and Saccharomyces cerevisiae^41, 43^. The values of *n*_ssc_ of all the networks belonging to the two types are higher than 0.9, which are substantially larger than the mean value of *n*_ssc_ of other networks. Consistent with the results of structural edge controllability, we find that regulatory networks are not only well-controllable under the edge dynamics but their controllability is also robust against uncertainties and variations in the interaction strengths. Except for the two types of networks, the effect of interaction strengths among the edges can be significant in other real world networks. For those networks, it is necessary to consider interaction strengths, in addition to the network structure, to gain insights into the edge controllability, especially when the networks are undirected.

**Table 3:** Controllability of edge dynamics in real networks.

Type	No.	class	Name	*N*	*M*	${{\bf{n}}}_{{\bf{D}}}^{{\bf{U}}}$	${{\bf{n}}}_{{\bf{D}}}^{{\bf{L}}}$	${{\bf{m}}}_{{\bf{D}}}^{{\bf{U}}}$	${{\bf{m}}}_{{\bf{D}}}^{{\bf{L}}}$	*n*_*SSC*_
Regulatory	1	directed	Ownership-USCorp	8497	6726	0.140	0.136	0.938	0.924	0.992
	2	directed	TRN-EC-2	423	578	0.246	0.220	0.879	0.829	0.946
	3	directed	TRN-Yeast-1	4684	15451	0.058	0.052	0.984	0.947	0.963
	4	directed	TRN-Yeast-2	688	1079	0.180	0.177	0.968	0.952	0.983
Trust	5	directed	Prison inmate	67	182	0.821	0.403	0.692	0.319	0.478
Food Web	6	directed	St.Marks	45	224	0.689	0.533	0.835	0.563	0.556
	7	directed	Seagrass	49	226	0.694	0.449	0.827	0.518	0.510
	8	directed	Grassland	88	137	0.330	0.318	0.620	0.606	0.977
	9	directed	Ythan	135	601	0.467	0.304	0.864	0.597	0.756
	10	directed	Silwood	154	370	0.208	0.188	0.897	0.797	0.942
	11	directed	Little Rock	183	2494	0.989	0.639	0.927	0.603	0.508
Electronic circuits	12	directed	S208a	122	189	0.541	0.451	0.413	0.344	0.910
	13	directed	s420a	252	399	0.556	0.456	0.416	0.348	0.901
	14	directed	s838a	512	819	0.563	0.459	0.418	0.350	0.896
Neuronal	15	directed	C. elegans	297	2359	0.909	0.549	0.886	0.374	0.253
Citation	16	directed	Small World	233	1988	0.300	0.210	0.902	0.729	0.738
	17	directed	SciMet	2729	10416	0.525	0.360	0.862	0.623	0.689
	18	directed	Kohonen	3772	12731	0.343	0.230	0.877	0.715	0.779
Internet	19	directed	Political blogs	1224	19090	0.819	0.619	0.956	0.525	0.472
	20	directed	p2p-1	10876	39994	0.380	0.334	0.877	0.591	0.715
	21	directed	p2p-2	8846	31839	0.376	0.344	0.883	0.628	0.732
	22	directed	p2p-3	8717	31525	0.376	0.343	0.884	0.625	0.726
Organizational	23	directed	Freeman-1	34	695	1	0.353	0.951	0.111	0
	24	directed	Consulting	46	879	1	0.522	0.950	0.150	0.065
Language	25	directed	English words	7381	46281	0.479	0.158	0.862	0.210	0.566
	26	directed	French words	8325	24295	0.329	0.157	0.736	0.216	0.747
Transportation	27	directed	USair97	332	2126	0.681	0.437	0.894	0.400	0.557
	28	undirected	USA top-500	500	2980	0.850	1/*N*	0.916	1/2*M*	0.150
Social communication	29	undirected	Facebook	4039	88234	0.981	1/*N*	0.977	1/2*M*	0.019
Internet	30	undirected	Internet-1997	3015	5156	0.522	1/*N*	0.708	1/2*M*	0.478
	31	undirected	Internet-1999	5357	10328	0.635	1/*N*	0.741	1/2*M*	0.365
	32	undirected	Internet-2001	10515	21455	0.648	1/*N*	0.755	1/2*M*	0.352
Autonomous systems	33	undirected	Oregon1-010331	10670	22002	0.651	1/*N*	0.758	1/2*M*	0.349
	34	undirected	Oregon1-010526	11174	23409	0.654	1/*N*	0.761	1/2*M*	0.346
	35	undirected	Oregon2-010331	10900	31180	0.704	1/*N*	0.825	1/2*M*	0.296
	36	undirected	Oregon2-010526	11461	32730	0.712	1/*N*	0.825	1/2*M*	0.289
	37	undirected	AS-733	6474	13895	0.645	1/*N*	0.767	1/2*M*	0.355
Collaboration networks	38	undirected	Ca-GrQc	5242	14496	0.806	0.068	0.825	0.012	0.228
	39	undirected	Ca-HepTh	9877	25998	0.813	0.043	0.815	0.008	0.214
	40	undirected	Ca-HepPh	12008	118521	0.889	0.023	0.950	0.001	0.124
	41	undirected	Ca-AstroPh	18772	198110	0.939	0.015	0.953	0.001	0.068

For each network, its type, class, name, number *N* of nodes, number *M* of edges, the upper bounds (${n}_{{\rm{D}}}^{{\rm{U}}}$ and ${m}_{{\rm{D}}}^{{\rm{U}}}$), the lower bounds (${n}_{{\rm{D}}}^{{\rm{L}}}$ and ${m}_{{\rm{D}}}^{{\rm{L}}}$), and strong structural controllability measure *n*_ssc_ are shown. For data sources and references, see Supplementary Table S2.

Another finding for real-world networks is that the values of the lower bound ${n}_{{\rm{D}}}^{{\rm{L}}}$ for undirected networks are much smaller than those for directed networks, in accordance with the results from the model networks. This indicates that real undirected networks have a much higher potential to be optimized for control through adjusting the interaction strengths. However, the values of *n*_ssc_ for undirected networks are generally lower than those for directed networks, suggesting that undirected networks are more sensitive to perturbations of the interaction strengths. A trade-off thus exists between the controllability and sensitivity for undirected networks. The correspondence of the value of *n*_ssc_ to the gap size between the upper and lower bounds in model networks also exists for real-world networks: a higher (lower) value of *n*_ssc_ is usually associated with a smaller (larger) gap, as shown in Table 3.

**Figure 4:**
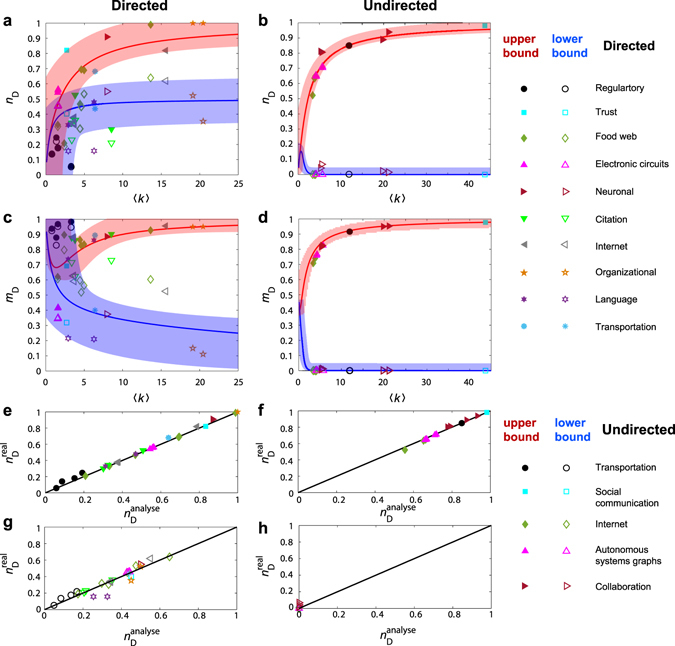
Controllability bounds of real-world networks. For a number of real-world directed and undirected networks, (**a**,**b**) upper and lower bounds of *n*_D_, respectively, (**c**,**d**) upper and lower bounds of *m*_D_, respectively, (**e**,**f**) numerically obtained upper bound ${n}_{{\rm{D}}}^{{\rm{real}}}$ and the theoretical prediction ${n}_{{\rm{D}}}^{{\rm{analyse}}}$, respectively, and (**g**,**h**) numerically obtained lower bound ${n}_{{\rm{D}}}^{{\rm{real}}}$ and the theoretical prediction, respectively. The curves of the upper and lower bounds in (**a**–**d**) are analytical results of model networks with an exponential degree distribution (**a**) and with a power-law degree distribution (**b**–**d**) (see Supplementary Notes 5 and 8 for the analytical derivations).

We also study the dependence of the controllability bounds of the real-world networks on the average degree 〈*k*〉. As shown in Fig. 4(a–d), the values of ${n}_{{\rm{D}}}^{{\rm{U}}}$ and ${n}_{{\rm{D}}}^{{\rm{L}}}$ for real-world networks with different values of 〈*k*〉 indeed constitute the universal controllability bounds. Due to the small influence of network topology on controllability (Fig. 3), the values of ${n}_{{\rm{D}}}^{{\rm{U}}}$ and ${n}_{{\rm{D}}}^{{\rm{L}}}$ of real directed networks tend to spread in two relatively small regions, which is analogous to the behavior in model networks (Fig. 3). In contrast, the influence of the network topology on the bounds of undirected networks is much weaker than for directed networks, leading to much smaller variance about the theoretical estimation. Given the degree distribution, the controllability bounds can be predicted more precisely by eliminating the effect of the degree distribution. For each network, a good agreement is obtained between the analytical prediction and the simulation results, as shown in Fig. 4(e–h).

**Figure 5:**
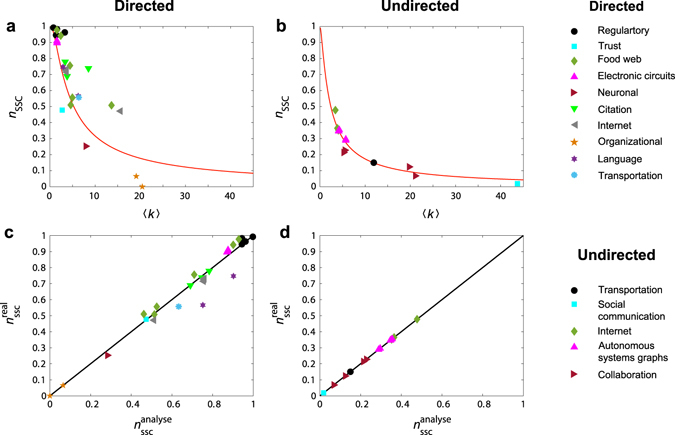
Strong structural controllability of real-world networks. (**a**,**b**) Strong structural controllability measure *n*_ssc_ as a function of the average degree 〈*k*〉 for real directed and undirected networks, respectively. (**c**,**d**) Numerically calculated ${n}_{{\rm{ssc}}}^{{\rm{real}}}$ and theoretical prediction ${n}_{{\rm{ssc}}}^{{\rm{analyse}}}$, respectively. In (**a**,**b**), the curves represent analytical results of a model network with an exponential degree distribution (see Supplementary Notes 7 and 8 for the analytical derivations).

Figure 5 shows the dependence of *n*_scc_ on 〈*k*〉 for real networks. We find that the values of *n*_scc_ are primarily determined by 〈*k*〉, especially for real undirected networks, in which the degree distribution has an insignificant effect on *n*_scc_. In fact, the role played by the distribution is marginal in our theoretical prediction of *n*_scc_ for model networks with an exponential degree distribution. Nonetheless, for real-world networks, their degree distribution can be taken into account and we obtain a reasonable agreement between the theoretically predicted and numerical values of *n*_scc_ for both directed and undirected networks (see Supplementary Fig. S5 for *m*_D_ and Note 8 for the analytical results). We find that the effect of degree distribution on *n*_ssc_ is quite similar to that on the controllability bounds.

## Discussion

Most existing frameworks of controllability of complex networks focus on nodal dynamical processes. In real-world networks, however, edge dynamics can also be important, such as the Internet, transportation and modern social networks. Network controllability based on edge dynamics was first addressed by Nepusz and Vicsek^38^, who introduced a mathematical framework to describe generic edge processes through switchboard dynamics, and uncovered significant differences between network nodal and edge controllability. However, their framework, being fundamentally a structural controllability framework, was limited to directed networks. The goal of our work is to develop an edge controllability framework that is universally applicable to all types of complex networks, directed or undirected, weighted (with arbitrary distributions) or unweighted. To accomplish this goal, we propose a class of generalized switchboard dynamics and exploit the exact controllability theory^21^ for complex networks.

Comprehensive mathematical analyses and extensive numerical tests with model and real-world networks have revealed a number of striking properties associated with edge controllability. For example, it is exclusively determined by the local topology and the interaction strengths, and this holds generally for arbitrary networks with any distributions of interaction strengths. This result provides a unique configuration of driver nodes, in stark contrast to what the nodal controllability theory can offer where it usually yields many configurations of the driver nodes. More strikingly, our framework is capable of providing a unique configuration of SSC nodes, a notion that has significant applied value for network control, whereas no structural controllability theory is able to offer a criterion for determining the SSC nodes. Our framework also allows us to address the challenging issue of upper and lower controllability bounds, and we prove that only given the local topology of each node, the controllability bounds of each node and those of the whole network can be completely determined. This result enables analytical predictions of the bounds and the SSC property based solely on the degree distribution. We also prove that the upper and lower bounds correspond to unweighted and structural switching matrices, respectively. A finding is that, for a fully SSC network, the gap between these bounds must vanish. In general, the interaction strengths play a more significant role in edge controllability than the network structure does, due to the typically large range between the controllability bounds, where an arbitrary degree of controllability in between the bounds can be achieved by adjusting the interaction strengths.

Applying our universal edge controllability framework to a large number of real-world networks, we find that transcriptional regulatory networks and electronic circuits possess the highest strong structural controllability. We also find that directed networks in the real world tend to be more SSC than undirected networks, and that sparser networks are usually more SSC than denser networks.

Our work raises a number of open questions, the answers to which would further deepen our understanding of the controllability of real world complex networks. For example, can a method be develop to implement target control for the general edge dynamics? What is the effect of the correlation between in-degrees and out-degrees on the edge controllability? Would it be possible to realize partial control of a subset of edges from a minimum number of driver nodes? Are there any approaches to optimize edge controllability through small perturbation? What is the energy cost in controlling general edge dynamics? Is it possible to treat nonlinear edge dynamics? Taken together, the framework developed here provides a base to address these questions, opening a new avenue towards fully controlling real networked systems in a wide range of fields.

## Methods

### Relationship between switching matrix *S*_*v*_ and adjacency matrix *W* for line graphs

In GSBD, all nonzero elements in the adjacency matrix *W* of the line graph *L*(*G*) come from the switching matrices in the original directed or undirected network *G*, and the nonzero elements in the identical columns (rows) stem from the same switching matrix. An example is shown in Fig. 1, where the nonzero elements in *W* of *L*(*G*) (Fig. 1(d,g)) correspond to the elements with the same color in the switching matrices of the original network (Fig. 1(c,f)). Furthermore, according to the generic property of directed line graphs, any two columns (rows) of the adjacency matrix *W* are either identical or orthogonal to each other. As a result, the contribution of a switching matrix *S*_*v*_ to the rank of *W* is the rank of *S*_*v*_, leading to the following relation between the ranks of the switching matrices and the adjacency matrix:

7 $${\rm{rank}}(W)=\sum _{i=1}^{N}{\rm{rank}}({S}_{i}),$$

where *N* is the number of nodes in the original network *G*, and the relation is rigorous for any networks (see Supplementary Note 2). The relation in combination with the exact controllability theory^21^ leads to our general formulas for *M*_D_ (Eq. (3)) and *N*_D_ (Eq. (5)).

### Controllability bounds of nodes and edges

The upper and lower bounds of controllability exist generally in a network. They can also be defined for each node in the network, as determined by the interaction strengths between the incoming and outgoing edges of the node. Because controllability is determined by the switching matrix of each node, we can ascertain the existence of the upper and lower bounds for any network by proving the existence of the bounds for each node. Specifically, for a node with ${k}_{v}^{+} > 0$ and ${k}_{v}^{-} > 0$, the maximum rank of its switching matrix is equal to the smaller value of the numbers of rows (${k}_{v}^{+}$) and columns (${k}_{v}^{-}$), which corresponds to that of the structural switching matrix. In addition, the minimum rank of the switching matrix is *S*_*v*_ = 1 if all rows (columns) are linearly dependent, which can be achieved in an unweighted switching matrix with identical element values. Moreover, the upper and lower bounds of a network can be reached if the corresponding bounds of every node are realized. We can thus conclude that the upper and lower bounds of *M*_D_ and *N*_D_ of a network correspond to unweighted and structural switching matrices, respectively.

### Driven edges and driver nodes associated with multiple network components

In a network with a single connected component, the driver nodes and the driven edges associated with the upper and lower bounds can be identified based on local topological information of nodes. An extreme case is where all nodes and edges are non-essential. In this case, we can randomly select a node to be a driver node on which an input signal is imposed, and the driver node is determined by the second term in Equation (5). In most cases, the contribution of the second term for a single connected component is negligible, compared with that of the first term. For a network consisting of a number of isolated components, the effect of each connected component on *N*_D_ must be taken into account, especially for undirected networks. In particular, for an undirected network with structural switching matrices, each connected component requires a driver node because every node is non-essential in the component. In this case, the second term in Equation (5) cannot be neglected. Table 1 lists the complete formulas of *N*_D_ and *M*_D_ in terms of both local topology and multiple components.

### Identification of SSC nodes

A node is either SSC or weakly structurally controllable (WSC). For a WSC node, element values in its switching matrix *S*_*v*_ will affect the rank of *S*_*v*_ and the category that the node belongs to. For an SSC node, its lower and upper bounds coincide with each other so that its category is determined only by its local structure. As a result, whether a node is SSC can be discerned in terms of its in- and out-degrees.

More specifically, for a node with ${k}_{v}^{+}=0$ or ${k}_{v}^{-}=0$, there is no switching matrix and the node is SSC. For a node with ${k}_{v}^{+} > 0$ and ${k}_{v}^{-} > 0$, the minimum rank of its *S*_*v*_ is one. If ${k}_{v}^{+}=1$ or ${k}_{v}^{-}=1$, any change in the value of the nonzero elements will not affect the rank of *S*_*v*_, because *S*_*v*_ is always unity, i.e., the minimum rank. Thus, in this case, the category to which the node belongs will not be affected by the element values in *S*_*v*_. For a node with ${k}_{v}^{+} > 1$ and ${k}_{v}^{-} > 1$, the possible maximum and minimum ranks of *S*_*v*_ are typically different, rendering the node WSC. Taken together, a node is SSC if and only if ${k}_{v}^{+}\le 1$ or ${k}_{v}^{-}\le 1$ in a directed network and *k*_*v*_ ≤ 1 in an undirected network. (For the special case of a single component composed of non-essential nodes, the criterion for identifying any SSC node is the same).

### Networks analyzed

The model networks used in this paper are the Erdös-Rényi random networks^44^ and scale-free networks^45^. How to generate model networks is provided in Supplementary Note 9. The real networks are described in Supplementary Table S2.

## Electronic supplementary material

Supplementary Information
