# Supplementary material for: Universal framework for edge controllability of complex networks
Source: Sci Rep. 2017 Jun 26;7:4224. doi: 10.1038/s41598-017-04463-5 (PMC5484715; doi:10.1038/s41598-017-04463-5)
Supplement: Supplementary file 1 — Supplementary Information [file 41598_2017_4463_MOESM1_ESM.pdf]

Supplementary Figures

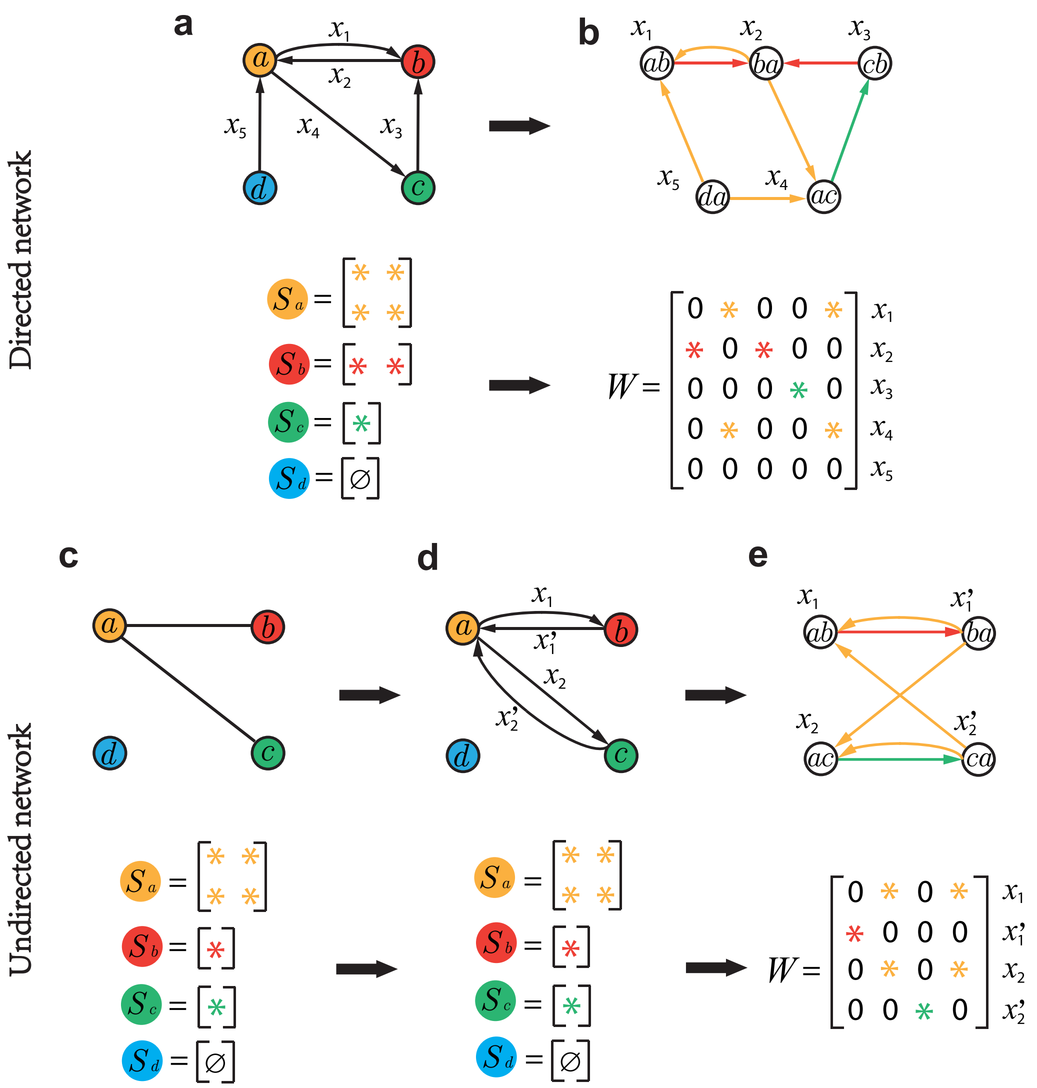

Supplementary Figure S1 | General switchboard dynamics. (**a**) A directed graph G with four nodes a, b, c, and d, five edges x_i ($i = 1, \dots, 5$), and its switching matrices S_a, S_b, S_c and S_d corresponding to the nodes with the same colours. (**b**) The line graph $L(G)$ of the original directed graph G. The colours of the edges in $L(G)$ corresponds to that of the nodes in (a). The nonzero elements in the adjacent matrix of $L(G)$ correspond to the elements with same colours in the switching matrices of G in (a). (**c**) An undirected graph G with four nodes a, b, c, and d, two edges, and its switching matrices S_a, S_b, S_c and S_d corresponding to the nodes with same colours. (**d**) The bidirectional (undirected) graph transformed from G by turning each edge in G to two directed edges with opposite directions, i.e., (x_i, x_i') ($i = 1, 2$). Its switching matrices are the same as that in (c). (**e**) The line graph $L(G)$ of the bidirectional (undirected) graph. The colours of the edges in $L(G)$ corresponds to that of the nodes in (d). The nonzero elements in the adjacent matrix of $L(G)$ are corresponding to the elements with same colours in the switching matrices of G in (c) and the bidirectional (undirected) graph in (d).

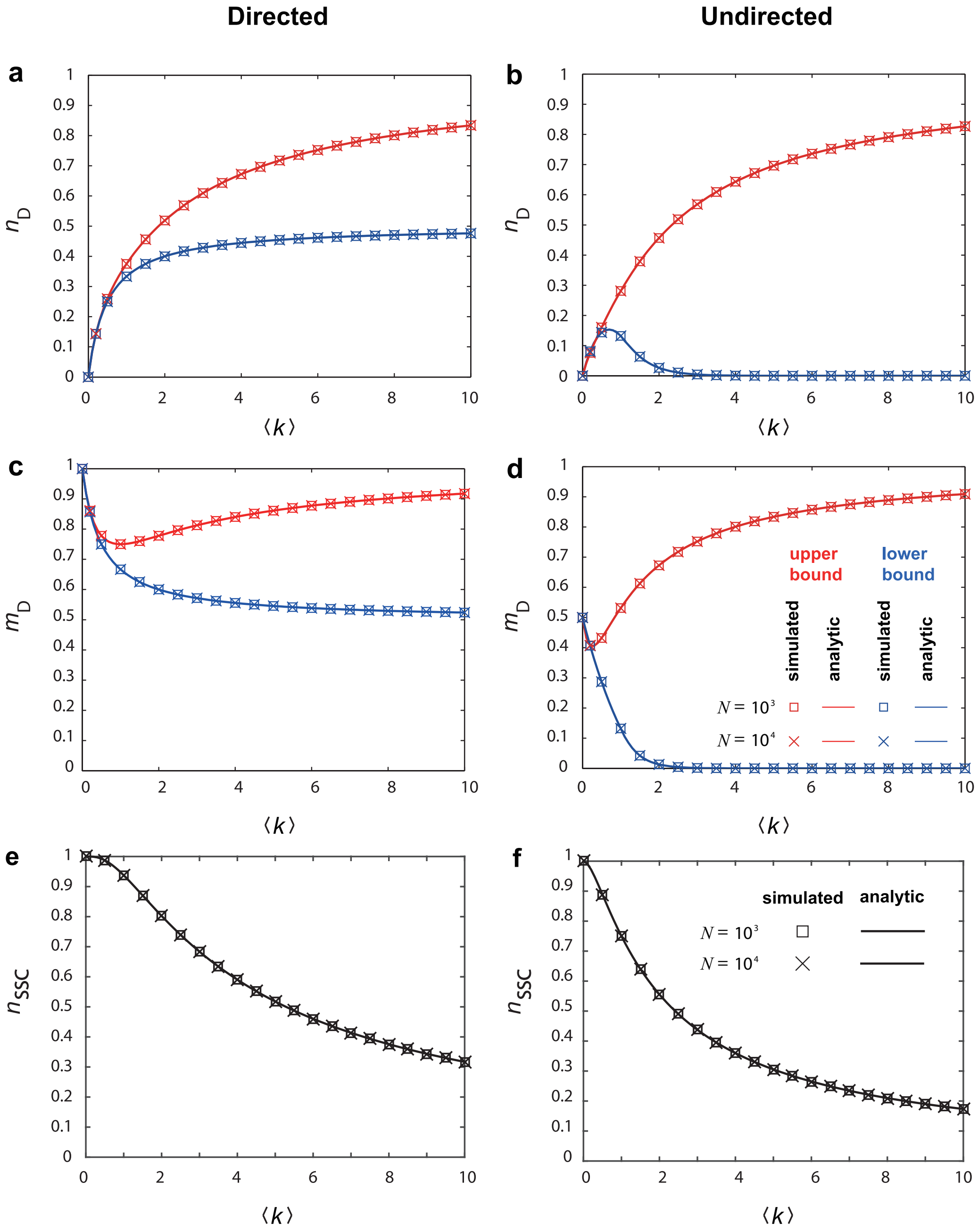

Supplementary Figure S2 | **Controllability bounds and strong structural controllability of exponentially distributed networks.** (**a**)-(**b**) The upper and lower bounds of $n_{\rm D}$ for directed and undirected networks with exponential degree distributions (EX). (**c**)-(**d**) The upper and lower bounds of $m_{\rm D}$ for directed and undirected EX networks. (**e**)-(**f**) Strong structural controllability $n_{\rm ssc}$ as a function of the average degree $\langle k \rangle$ for directed and undirected EX networks. Data points are numerical results and curves are analytical results. All the numerical results are obtained by averaging over 50 independent networks realizations. See Supplementary Note 10 for network models.

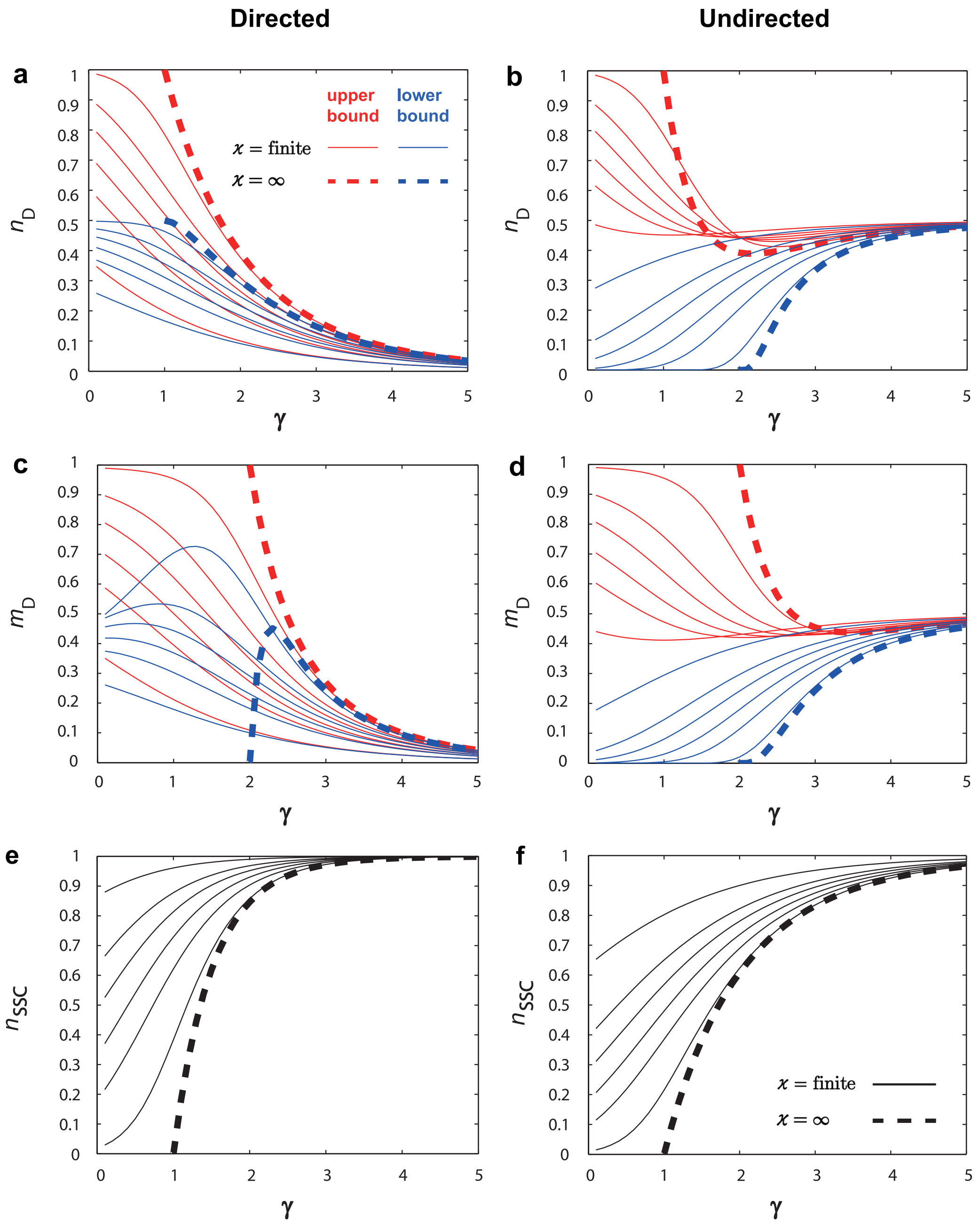

Supplementary Figure S3 | Controllability bounds and strong structural controllability of Scale-free networks from configuration model. (**a**)-(**b**) The upper and lower bounds of $n_{\rm D}$ for directed and undirected SF networks. (**c**)-(**d**) The upper and lower bounds of $m_{\rm D}$ for directed and undirected SF networks. (**e**)-(**f**) Strong structural controllability $n_{\rm ssc}$ as a function of the scaling exponent γ for directed and undirected SF networks. γ is the scaling exponent and κ is the exponential cutoff parameter of the SF network. The exponential cutoff parameter for the blue full lines in (b) and (d) and the black full lines in (e) and (f) are, from top to bottom, $\kappa = 1, 2, 3, 5, 10, 100$, and reverse order for others. The dashed lines are associated with $\kappa = \infty$. Note that the range of the upper or lower bounds is large in the SF networks generated by configuration model. This is because of the fact that the average degree $\langle k \rangle$ depends on the γ and κ. Along with the change of γ and κ, $\langle k \rangle$ changes as well. Due to the significant role of $\langle k \rangle$ in controllability, large variance of controllability is observed by changing γ and κ. See Supplementary Note 10 for network models.

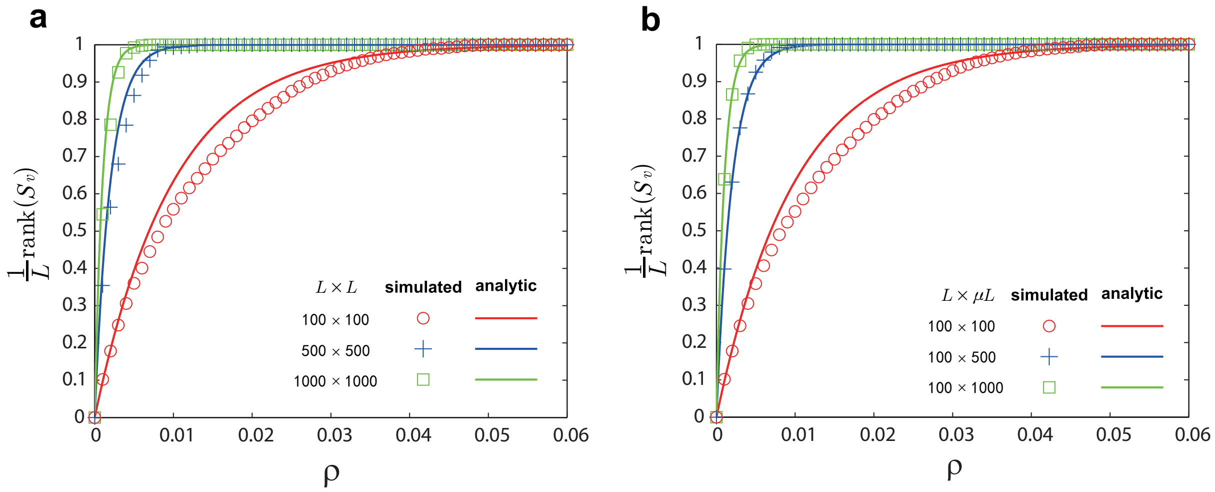

Supplementary Figure S4 | Transition between upper and lower bounds. The transition from the upper bound ($\frac{1}{L}\mathrm{rank}(S_v) = 1/L$) to the lower bound ($\frac{1}{L}\mathrm{rank}(S_v) = 1$) by adjusting the density ρ of the elements with random value in (**a**) $L \times L$ switching matrix and (**b**) $L \times \mu L$ switching matrix. Data points are numerical results and curves are analytical results. All the numerical results are obtained by averaging over 50 independent networks realizations.

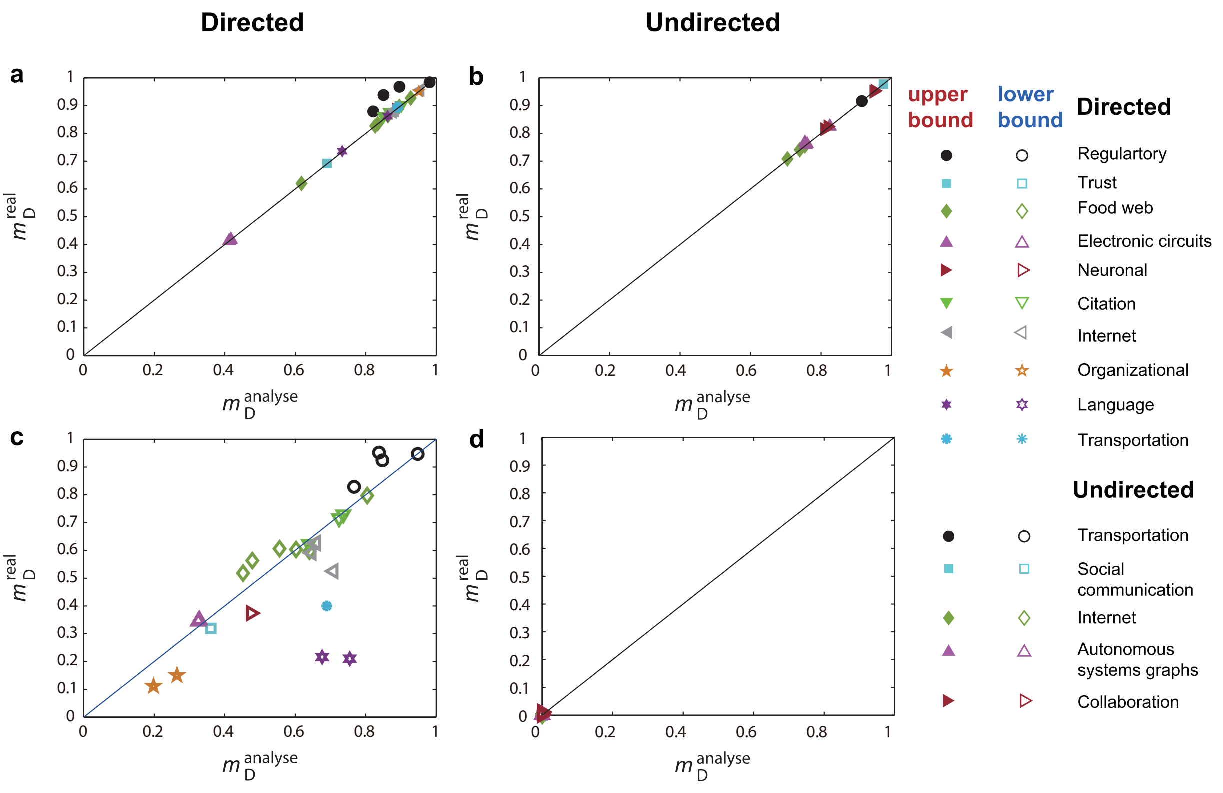

Supplementary Figure S5 | Controllability bounds of driven edges in real networks. (**a**)-(**b**) The upper bound $m_{\mathrm{D}}^{\mathrm{real}}$ obtained directly and the theoretical prediction of the upper bound $m_{\mathrm{D}}^{\mathrm{analyse}}$ in the real directed and undirected networks, respectively. (**c**)-(**d**) The lower bound $m_{\mathrm{D}}^{\mathrm{real}}$ obtained directly and the theoretical prediction of the lower bound $m_{\mathrm{D}}^{\mathrm{analyse}}$ in real directed and undirected networks, respectively. See Supplementary Note 8 for the analytical results of the real networks.

Supplementary Tables

Supplementary Table S1 | Controllability of edge dynamics in regular networks. For each regular network with nodes number N, we show the number of its directed edges M, the upper bounds ($N_{\mathrm{D}}^{\mathrm{U}}$ and $M_{\mathrm{D}}^{\mathrm{U}}$), the lower bounds ($N_{\mathrm{D}}^{\mathrm{L}}$ and $M_{\mathrm{D}}^{\mathrm{L}}$) and strong structural controllability N_{ssc}.

Name	M	$N_{\mathrm{D}}^{\mathrm{U}}$	$N_{\mathrm{D}}^{\mathrm{L}}$	$M_{\mathrm{D}}^{\mathrm{U}}$	$M_{\mathrm{D}}^{\mathrm{L}}$	N_{SSC}
Directed chain graph	$N-1$	1	1	1	1	N
Undirected chain graph	$2(N-1)$	$N-2$	1	$N-2$	1	2
Directed ring graph	N	1	1	1	1	N
Undirected ring graph	$2N$	N	1	N	1	0
Directed star graph with the central point $k_v^+ = N-1$	$N-1$	1	1	$N-1$	$N-1$	$N-1$
Directed star graph with the central point $k_v^- = N-1$	$N-1$	$N-1$	$N-1$	$N-1$	$N-1$	$N-1$
Undirected star graph	$2(N-1)$	1	1	$N-2$	1	$N-1$
Directed fully connected graph	$N(N-1)$	N	1	N^2-2N	1	0
Undirected fully connected graph	$N(N-1)$	N	1	N^2-2N	1	0
Directed k-regular connected graph ($k_v^+ = k_v^- = k/2$ for each node) with $k>2$	$Nk/2$	N	1	$N(k/2-1)$	1	0
Undirected k-regular connected graph ($k_v = k$ for each node) with $k>1$	Nk	N	1	$N(k-1)$	1	0

Supplementary Table S2 | Summary of the real directed and undirected networks analyzed in the paper. For each real network, we show the number of nodes, the number of edges, physical description and the semantics of edges.

Type	No.	Class	Name	Nodes	Edges	Description (semantics of $A \to B$)
Regulatory	1	Directed	Ownership-USCorp [42]	8497	6726	Ownership network of US corporations (A owns B)
	2	Directed	TRN-EC-2 [41]	423	578	Transcriptional regulatory network (A regulates B)
	3	Directed	TRN-Yeast-1 [43]	4684	15451	Same as above
	4	Directed	TRN-Yeast-2 [41]	688	1079	Same as above
Trust	5	Directed	Prison inmate [56, 57]	67	182	Social networks of positive sentiment (A is trusted by B).
Food Web	6	Directed	St.Marks [58]	45	224	Food Web in YthanEstuary (A preys on B).
	7	Directed	Seagrass [59]	49	226	Food Web in Seagrass (A preys on B).
	8	Directed	Grassland [60]	88	137	Food Web in Grassland (A preys on B).
	9	Directed	Ythan [60]	135	601	Food Web in Ythan (A preys on B).
	10	Directed	Silwood [61]	154	370	Food Web in Silwood (A preys on B).
	11	Directed	Little Rock [62]	183	2494	Food Web in Littlerock (A preys on B).
Electronic circuits	12	Directed	S208a [41]	122	189	Electronic sequential logic circuit (B is a function of A).
	13	Directed	s420a [41]	252	399	Same as above.
	14	Directed	s838a [41]	512	819	Same as above.
Neuronal	15	Directed	C. elegans [63]	297	2359	Neural network of C.elegans (B is within one synapse or gap junction distance from A).
Citation	16	Directed	Small World [64]	233	1988	Citation network in S.Milgram's Small World (A cites B).
	17	Directed	SciMet [64]	2729	10416	Citation network in Scientometrics (A cites B).
	18	Directed	Kohonen [65]	3772	12731	Citation network in T.Kohonen's Small World (A cites B).
Internet	19	Directed	Political blogs [66]	1224	19090	Hyper links between web logs on US politics (A links to B).
	20	Directed	p2p-1 [67, 68]	10876	39994	Gnutella peer-to-peer file sharing network (A sent messages to B).
	21	Directed	p2p-2 [67, 68]	8846	31839	Same as above.
	22	Directed	p2p-3 [67, 68]	8717	31525	Same as above.
Organizational	23	Directed	Freeman-1 [69]	34	695	Social network of network researchers (A was nominated by B on a questionnaire as acquaintance).
	24	Directed	Consulting [70]	46	879	Social network from a consulting company (B turned to A for advice).
Language	25	Directed	English words [71]	7381	46281	The words network in English (A links to B).
	26	Directed	French words [71]	8325	24295	The words network in French (A links to B).
Transportation	27	Directed	USair97 [72]	332	2126	US Airline 1997 (There are flights from A to B).
	28	Undirected	USA top-500 [73]	500	5980	Flight network in USA.
Social communication	29	Undirected	Facebook[74]	4039	88234	The online social network as similar as Facebook.
Internet	30	Undirected	Internet-1997 [75]	3015	5156	Autonomous Systems topology of the Internet.
	31	Undirected	Internet-1999 [75]	5357	10328	Same as above.
	32	Undirected	Internet-2001 [75]	10515	21455	Same as above.
Autonomous systems	33	Undirected	Oregon1-010331 [67]	10670	22002	Autonomous Systems peering information inferred from Oregon route-views.
	34	Undirected	Oregon1-010526 [67]	11174	23409	Same as above.
	35	Undirected	Oregon2-010331 [67]	10900	31180	Same as above.
	36	Undirected	Oregon2-010526 [67]	11461	32730	Same as above.
	37	Undirected	AS-733 [67]	6474	13895	Same as above.
Collaboration networks	38	Undirected	Ca-GrQc[67]	5242	14496	Collaboration network of Arxiv General Relativity.
	39	Undirected	Ca-HepTh [67]	9877	25998	Collaboration network of Arxiv High Energy Physics Theory.
	40	Undirected	Ca-HepPh [67]	12008	118521	Collaboration network of Arxiv High Energy Physics.
	41	Undirected	Ca-AstroPh [67]	18772	198110	Collaboration network of Arxiv Astro Physics.

Supplementary Note 1: The minimum number of driver node in a line graph

Exact controllability. The exact controllability was recently proposed in Ref. [22] as a framework for identifying the minimum set of driver nodes to achieve full control of networks with arbitrary structures and link weights between nodes. Consider a network with N nodes described by the ordinary differential equation [46, 47]:

$$\dot{\mathbf{x}} = A\mathbf{x} + B\mathbf{u}, \tag{S1}$$

where vector $\mathbf{x} = (x_1, x_2, ..., x_N)^T$ captures the states of nodes, $\mathbf{u}$ represents the external input signals with $\mathbf{u} = (u_1, u_2, ..., u_m)^T$, $A \in \mathbb{R}^{N\times N}$ denotes the coupling matrix among nodes with a_{ij} representing the weight of a directed link from node j to i (for undirected, $a_{ij} = a_{ji}$), and $B \in \mathbb{R}^{N\times m}$ is the control matrix.

The exact controllability theory for complex networks stems from the Popov-Belevitch-Hautus (PBH) test [46, 47, 48]. According to the PBH test, system (S1) is fully controllable if and only if

$$\mathrm{rank}\,(cI_N - A, B) = N \tag{S2}$$

for any complex number c, where I_N is the identity matrix of dimension N. The minimum number N_{D} of driver nodes is defined in terms of B as $N_{\mathrm{D}} = \min\{\mathrm{rank}(B)\}$.

The rank of the matrix $[cI_N - A, B]$ is contributed by the number of its linearly independent rows. Thus, the minimum rank of B should be equal to the number of linearly dependent rows in matrix $[cI_N - A]$, which is equal to $N - \mathrm{rank}(\lambda^{\mathrm{M}} I_N - A)$, where λ^{M} is the eigenvalue associated with the maximum geometric multiplicity. Note that $N - \mathrm{rank}(\lambda^{\mathrm{M}} I_N - A)$ is nothing but the maximum geometric multiplicity of the state matrix A. Thus, for arbitrary network structures and link weights, the minimum number N_{D} of driver nodes is determined by the maximum geometric multiplicity $\mu(\lambda_i)$ of the eigenvalue λ_i of A:

$$N_{\mathrm{D}} = \max_i\{\mu(\lambda_i)\}. \tag{S3}$$

Particularly, for a large sparse network in the absence of self-loops, the expectation of eigenvalues of adjacent matrix is $E(\lambda) = 1/N \sum_{i=1}^{N} \lambda_i = 1/N \sum_{i=1}^{N} a_{ii} \approx 0$. Thus, $\mu(\lambda_i^{\mathrm{M}})$ arises at $\lambda = 0$ with high probability [49]. Furthermore, we know that the geometric multiplicity associated with the zero eigenvalue is equal to rank deficiency [50]: $\mu(0) = N - \mathrm{rank}(A)$. Thus, the minimum number N_{D} of driver nodes determined by the maximum geometric multiplicity of $\lambda = 0$ in large sparse networks is

$$N_{\mathrm{D}} = \max\{1, N - \mathrm{rank}(A)\}. \tag{S4}$$

The minimum number of driver nodes in a line graph. For the dynamical process occurring on the node of a line graph $L(G)$, according to the exact controllability theory [22], the minimum number $N_{\mathrm{D}}^{\mathrm{L(G)}}$ of driver nodes in $L(G)$ is determined by the maximum geometric multiplicity of its adjacent matrix, i.e., $N_{\mathrm{D}}^{\mathrm{L(G)}} = \max_i\{\mu(\lambda_i)\}$. Now we focus on the expected eigenvalues in the line graph $L(G)$. We first estimate the sparsity of $L(G)$. The sparsity is defined by the ratio of the actual number of edges to the maximum number of possible edges among nodes. For a directed network, the sparsity is approximately $\rho = M/N^2$ for a sufficiently large N. Note that the number of nodes in the line graph $L(G)$ is equal to the number of edges in $L(G)$'s original directed or undirected network G. Hence the sparsity of $L(G)$ is

$$\begin{aligned}\rho_{\mathrm{L(G)}} = \frac{M_{\mathrm{L(G)}}}{N^2_{\mathrm{L(G)}}} &= \frac{\sum_{i=1}^N k_i^+ k_i^-}{[1/2\sum_{i=1}^N (k_i^+ + k_i^-)]^2} \\ &= \frac{\sum_{i=1}^N k_i^+ k_i^-}{1/4\sum_{i=1}^N \sum_{j=1}^N (k_i^+ k_j^+ + k_i^+ k_j^- + k_i^- k_j^+ + k_i^- k_j^-)},\end{aligned} \tag{S5}$$

where k_i^+ and k_i^- are the out-degree and in-degree of node i in G, respectively, and N is the number of nodes in G. This implies that in a line graph $L(G)$, the sparsity depends on the degree distribution of both in- and out-degree in G.

Consider a directed or undirected network G with homogeneous degree distribution. An extreme case is that each node has the same in- and out-degree. In this case, the sparsity of its line graph $L(G)$ is

$$\rho_{\mathrm{L(G)}} = \frac{M_{\mathrm{L(G)}}}{N^2_{\mathrm{L(G)}}} = \frac{Nk^2}{N^2k^2} = \frac{1}{N}, \tag{S6}$$

where k is the in- and out-degree of each node in G. Consider a directed or undirected network G with heterogeneous degree distribution. An extreme case is that one node v has $k_v^+ = k_v^- = N$ and the other $N-1$ nodes have $k_v^+ = k_v^- = 1$ in G. Then the sparsity of its line graph $L(G)$ is

$$\rho_{\mathrm{L(G)}} = \frac{M_{\mathrm{L(G)}}}{N^2_{\mathrm{L(G)}}} = \frac{N^2+N-1}{(2N-1)^2} \approx \frac{1}{4}. \tag{S7}$$

According to the above analysis, $L(G)$ of a homogeneous network G is sparse, so that zero dominates the eigenvalue spectrum and is associated with the maximum geometric multiplicity. Meanwhile, for the extreme case of the G with the most heterogeneous degree distribution, the adjacent matrix of its $L(G)$ is

$$W = \begin{bmatrix} & & * & & \\ & 0 & & \ddots & \\ & & & & * \\ * & \cdots & * & & \\ \vdots & \ddots & \vdots & 0 & \\ * & \cdots & * & & \end{bmatrix}, \tag{S8}$$

where W is a $(2N-1) \times (2N-1)$ matrix, and the nonzero elements in the lower-triangle stem from the $N \times N$ switching matrix of node v with $k_v^+ = k_v^- = N$ and other $N-1$ switching matrices contribute $N-1$ nonzero elements to the upper-triangle. Despite the violation of sparsity in this case, the expectation of eigenvalues of the adjacent matrix of $L(G)$ is still $E(\lambda) \approx 0$. Taken together, we demonstrate that for the line graph $L(G)$ of a large directed or undirected G with arbitrary structures, the maximum geometric multiplicity of its adjacent matrix occurs at the eigenvalue $\lambda = 0$. Thus, for an arbitrary line graph $L(G)$, its minimum number $N_{\mathrm{D}}^{\mathrm{L(G)}}$ of driver nodes is determined by the geometric multiplicity associated with the zero eigenvalue and can be evaluated by

$$N_{\mathrm{D}}^{\mathrm{L(G)}} = \max\{1, M - \mathrm{rank}(W)\}, \tag{S9}$$

where M is the number of edges in the original directed or undirected network G. We have examined that for all networks studied in the main text and in Supplementary Information, zero dominates eigenvalue spectrum of $L(G)$ and is associated with the maximum geometric multiplicity.

Supplementary Note 2: The relation between the switching matrix and the adjacency matrix of line graph.

For the relation between the switching matrix and the adjacency matrix of line graph, a lemma concerning the line graph is necessary, as follows [51]:

Lemma 1 *A directed network without multiple arcs is a line graph if and only if any two columns (rows) of its adjacent matrix are always either identical or orthogonal.*

In the adjacent matrix of the line graph $L(G)$, identical columns (rows) indicate that the locations of nonzero elements in two columns (rows) are the same, and orthogonal columns (rows) indicate that the locations of nonzero elements in two columns (rows) are different entirely. The above lemma indicates that, for two arbitrary nodes in the line graph, their neighbouring nodes are either the same or entirely different.

According to the definition of the general switchboard dynamics (GSBD) and the above lemma, we offer a theorem as follows.

Theorem 1 *In the GSBD, all of the nonzero elements in the adjacent matrix of line graph $L(G)$ stem from the switching matrices S_v in the original directed or undirected (bidirectional) graph G, and the nonzero elements in identical columns (rows) stem from the same switching matrix.*

Proof. For a line graph $L(G)$ stems from a directed or bidirectional network G, an edge in $L(G)$ represents a length-two directed path in the original network G, and each length-two directed path corresponds to an element in the switching matrix of a node in G. The key finding is that there is a one-to-one correspondence between the nonzero elements in the adjacent matrix of line graph $L(G)$ and the elements in the switching matrices of the original network G. Furthermore, according to the definition of the GSBD, we know that the value of a nonzero element in the adjacent matrix of $L(G)$ is equal to the value of the correspondent element in the switching matrix in the original network G. Thus, all the nonzero elements in the adjacent matrix of the line graph stem from the switching matrices in the original directed or undirected network.

In the GSBD, a switching matrix S_v is a $k_v^+ \times k_v^-$ matrix, and each element describes a switchboard relationship from one incoming edge to one outgoing edge of node v. According to the transformational rule, the nodes of $L(G)$ correspond to the edges of the original network G, and each edge of $L(G)$ represents a length-two directed path of G. Hence, a node v with $k_v^+ > 0$ and $k_v^- > 0$ in G contributes $k_v^+ + k_v^-$ nodes and $k_v^+ \times k_v^-$ edges to $L(G)$. Moreover, it is easy to see that, in the adjacent matrix W

of $L(G)$, the nonzero elements corresponding to the above $k_v^+ \times k_v^-$ edges locate in identical columns (rows). Taken together, the nonzero elements in the identical columns (rows) in the adjacent matrix of $L(G)$ stem from the same switching matrix in the original directed or undirected network G. This concludes our proof.

An example is shown in Fig. S1. For both directed and undirected (bidirectional) networks, the nonzero elements in the adjacent matrix of $L(G)$ correspond to the elements with the same colours in the switching matrices of G. We see that all nonzero elements in the adjacent matrix W of line graph $L(G)$ (Fig. S1(b) and (e)) stem from the switching matrices in the original directed or undirected (bidirectional) graph G (Fig. S1(a) and (c)), and the nonzero elements in identical columns (rows) stem from the same switching matrix.

Lemma 1 and Theorem 1 allow us to formulate the theorem below.

Theorem 2 *The rank of the adjacent matrix of line graph $L(G)$ is equal to the sum of the rank of all switching matrices in the original directed or undirected (bidirectional) network G, i.e.,*

$$\mathrm{rank}(W) = \sum_{i=1}^{N} \mathrm{rank}(S_i), \tag{S10}$$

where N is the number of nodes in the original directed or undirected network G.

Proof. According to Lamma 1 that any two columns (rows) in the adjacent matrix W of line graph $L(G)$ are always either identical or orthogonal, we can obtain the canonical by simply exchanging columns and rows (elementary transformation), which will not change $\mathrm{rank}(W)$. As an example, the adjacent matrix W in Fig. S1(b) can be transformed into the canonical form

$$W = \begin{bmatrix} * & * & 0 & 0 & 0 \\ * & * & 0 & 0 & 0 \\ 0 & 0 & * & * & 0 \\ 0 & 0 & 0 & 0 & * \\ 0 & 0 & 0 & 0 & 0 \end{bmatrix}. \tag{S11}$$

Because $\mathrm{rank}(W)$ is not changed, $\mathrm{rank}(W)$ is equal to the sum of the rank of sub-matrices composed of the nonzero elements in identical columns (rows). Moreover, according to Theorem 1, all nonzero elements in the adjacent matrix of $L(G)$ stem from the switching matrices in G, and the nonzero elements in identical columns (rows) stem from the same switching matrix. Therefore, the rank of a sub-matrix consisting of the nonzero elements in identical columns (rows) in the adjacent matrix of $L(G)$ is equal to the rank of its corresponding switching matrix in the original G. This concludes our proof.

Supplementary Note 3: The key results of N_{D} and M_{D}

Proof of the key results. In the GSBD, the dynamical process occurring in the edges of a directed or undirected G can be turned into the dynamical process on the nodes of G's line graph $L(G)$, and the driven edges in G correspond to the driver nodes in $L(G)$. Thus, the minimum number M_{D} of driven edges in a directed or undirected network G is equivalent to the minimum number $N_{\mathrm{D}}^{\mathrm{L(G)}}$ of driver nodes in the line graph $L(G)$.

We first consider $N_{\mathrm{D}}^{\mathrm{L(G)}}$ in a given line graph. For the line graph $L(G)$ of G with arbitrary structure, its $N_{\mathrm{D}}^{\mathrm{L(G)}}$ is determined by the geometric multiplicity associated with the zero eigenvalue, which can be estimated by Eq. (S9). Note that, in general, according to Eq. (S9), there is no driver node in a connected component if the adjacent matrix of this component is of full rank. However, for an isolated component, without driver node, external input signal cannot reach any node in the component, rendering the component uncontrollable. This thus calls for a single driver node that can be any single node in the component to receive input signals to fully control the component.

Therefore, we provide a general approach to identifying the minimum number M_{D} of driven edges in G, which is

$$M_{\mathrm{D}} = M - \sum_{i=1}^{N} \mathrm{rank}(S_i) + \sum_{i=1}^{C} \beta_i, \tag{S12}$$

where C is the number of connected components in G and $\beta_i = 1$ if the switching matrices of all nodes in component i are square matrices with full rank, and $\beta_i = 0$ otherwise.

Now we obtain the minimum number N_{D} of driver nodes required to control the dynamical process occurring in the edges of network G. We know that the edges number in G is equal to the sum of out-degree of each node, i.e., $M = \sum_{i=1}^{N} k_i^{+}$. Thus, Eq. (S12) can be reformulated to be

$$M_{\mathrm{D}} = \sum_{i=1}^{N} \big(k_i^{+} - \mathrm{rank}(S_i)\big) + \sum_{i=1}^{C} \beta_i. \tag{S13}$$

Equation (S13) indicates that a node i with $\mathrm{rank}(S_i) < k_i^{+}$ has to drive $k_i^{+} - \mathrm{rank}(S_i)$ outgoing edges of the node. To be specific, S_i of a driver node is not of full row-rank, which yields the general formula of N_{D}:

$$N_{\mathrm{D}} = N_{(\mathrm{rank}(S_i) < k_i^{+})} + \sum_{i=1}^{C} \beta_i, \tag{S14}$$

where β_i is the same as in Eq. (S12). Because a driver node can only drive its outgoing edges, we only consider the row rank of switching matrices rather than column rank. Therefore, despite $M = \sum_{i=1}^{N} k_i^{-}$, we only use $M = \sum_{i=1}^{N} k_i^{+}$ associated with outgoing links and the row rank of switching matrices,

which yields Eq. (S13). In the whole Supplementary Information below, rank refers to row-rank for simplicity.

N_{D} and M_{D} in the upper and lower bounds based on local structural information. We provide general formulas of N_{D} and M_{D} for both directed and undirected networks. The upper and lower bounds are associated with unweighted and structural switching matrices, respectively.

In general, for arbitrary switching matrices, $\mathrm{rank}(S_i)$ of all nodes has to be calculated to obtain N_{D} and M_{D}. However, for unweighted switching matrices and structural switching matrices, we are able to discern driver nodes and driven edges exclusively based on the in- and out-degrees of nodes.

For a directed network with unweighted switching matrices corresponding to the upper bound, the switching matrix of node v with $k_v^+ > 0$ and $k_v^- > 0$ can contribute only one to the rank of W, which accounts for the fact that a node with $k_v^+ > 1$ must be a driver node, and a node with $k_v^- = 0$ and $k_v^+ = 1$ must be a driver node as well. Thus for the upper bound, we have

$$N_{\mathrm{D}}^{\mathrm{DU}} = N_{(k_v^+>1)} + N_{(k_v^-=0,k_v^+=1)} + \sum_{i=1}^{C} \beta_i', \tag{S15}$$

where the number of nodes under some conditions is defined as $N_{(*)}$ with the conditions in its subscript and β_i' is 1 if the ith connected component contains only the nodes with $k_v^+ = k_v^- = 1$. The minimum number of driven edges is mainly determined by the number of nodes with $k_v^- > 0$ and $k_v^+ > 0$, i.e.,

$$M_{\mathrm{D}}^{\mathrm{DU}} = M - N_{(k_v^->0,k_v^+>0)} + \sum_{i=1}^{C} \beta_i'. \tag{S16}$$

For a directed network with structural switching matrices corresponding to the lower bound, the rank of the switching matrix of node v with $k_v^+ > 0$ and $k_v^- > 0$ contributes $\min(k_v^+, k_v^-)$ to the rank of W. Hence, a node with $k_v^- < k_v^+$ must be a driver node, and the minimum number of driver nodes is

$$N_{\mathrm{D}}^{\mathrm{DL}} = N_{(k_v^-<k_v^+)} + \sum_{i=1}^{C} \beta_i'', \tag{S17}$$

where β_i'' is 1 if the ith connected component is balanced (a connected component consisting of the nodes with $k_v^+ = k_v^- > 0$). The minimum number of driven edges is mainly determined by the sum of the smaller value between the in- and out-degree, which is

$$M_{\mathrm{D}}^{\mathrm{DL}} = M - \sum_{i=1}^{N} \min\{k_i^-, k_i^+\} + \sum_{i=1}^{C} \beta_i''. \tag{S18}$$

For an undirected (bidirectional) network with unweighted switching matrices corresponding to the upper bound, all nodes have the same in- and out-degree. We denote the degree of a node as $k_v = k_v^+ = k_v^-$. The minimum number of driver nodes is mainly determined by the nodes with $k_v > 1$, which is

$$N_{\mathrm{D}}^{\mathrm{BU}} = N_{(k_v>1)} + \sum_{i=1}^{C} \beta_i'. \tag{S19}$$

The minimum number of driven edges is mainly determined by the number of nodes with $k_v > 0$, which is

$$M_{\mathrm{D}}^{\mathrm{BU}} = M - N_{(k_v>0)} + \sum_{i=1}^{C} \beta_i'. \tag{S20}$$

For an undirected (bidirectional) network with structural switching matrices corresponding to the lower bound, all switching matrices are square matrices with full rank except the isolated nodes. A single driver node and a single driven edge are required in each balanced component. The minimum numbers of both driver nodes and driven edges are determined by the number of balanced components, which is

$$N_{\mathrm{D}}^{\mathrm{BL}} = M_{\mathrm{D}}^{\mathrm{BL}} = \sum_{i=1}^{C} \beta_i''. \tag{S21}$$

Supplementary Note 4: Reproducing structural edge controllability

Our GSBD framework can reproduce the structural edge controllability of directed networks. We will prove that N_{D} and M_{D} obtained by our framework in a directed network associated with structural switching matrices are equal to the structural edge controllability N_{D} and M_{D} in Ref. [38].

According to structural edge controllability [38], for a directed network with structural switching matrices, N_{D} is determined by the number of divergent nodes (the nodes with $k_v^+ > k_v^-$) in G and one arbitrary node from each balanced component, which is equal to $N_{\mathrm{D}}^{\mathrm{DL}}$ obtained from Eq. (S17).

Furthermore, according to structural edge controllability, each divergent node must control $k_v^+ - k_v^-$ of its outgoing edges, and the randomly selected nodes in each balanced component must control only one of its outgoing edges, which yields

$$\begin{aligned} M_{\mathrm{D}}^{\mathrm{SC}} &= \sum_{i=1}^{N} \max\left(k_i^+ - k_i^-, 0\right) + \sum_{i=1}^{C} \beta_i'' \\ &= \frac{1}{2}\sum_{i=1}^{N} \left|k_i^+ - k_i^-\right| + \sum_{i=1}^{C} \beta_i''. \end{aligned} \tag{S22}$$

In the following, we will prove that $M_{\mathrm{D}}^{\mathrm{DL}}$ obtained from Eq. (S18) is equal to the value of $M_{\mathrm{D}}^{\mathrm{SC}}$ calculated by the above equation. To be specific,

$$\begin{aligned} M_{\mathrm{D}}^{\mathrm{DL}} &= M - \sum_{i=1}^{N} \min\{k_i^+, k_i^-\} + \sum_{i=1}^{C} \beta_i'' \\ &= \frac{1}{2}\sum_{i=1}^{N} \left(k_i^+ + k_i^-\right) - \sum_{i=1}^{N} \min\{k_i^+, k_i^-\} + \sum_{i=1}^{C} \beta_i'' \\ &= \frac{1}{2}\sum_{i=1}^{N} \left[\left(k_i^+ + k_i^-\right) - 2\min\{k_i^+, k_i^-\}\right] + \sum_{i=1}^{C} \beta_i'', \end{aligned} \tag{S23}$$

where the parenthetical part is

$$\begin{aligned} &\left(k_i^+ + k_i^-\right) - 2\min\{k_i^+, k_i^-\} \\ =&\begin{cases} k_i^+ - k_i^- & \text{if } k_i^+ \geq k_i^- \\ k_i^- - k_i^+ & \text{if } k_i^+ < k_i^- \end{cases} \\ =&\left|k_i^+ - k_i^-\right|, \end{aligned} \tag{S24}$$

which proves $M_{\mathrm{D}}^{\mathrm{DL}} = M_{\mathrm{D}}^{\mathrm{SC}}$ for directed networks. Therefore, we prove that our GSBD framework reproduce the structural edge controllability in directed networks.

Supplementary Note 5: Analytical results

General formulas of $n_{\rm D}$ and $m_{\rm D}$ for arbitrary networks. The dependence of $n_{\rm D}$ and $m_{\rm D}$ in the upper and lower bounds on local structures allows us to derive analytical results of edge controllability in terms of the joint degree distribution of model networks. We assume that in- and out-degree of each node are uncorrelated to offer analytical results.

For a directed network with unweighted switching matrices associated with the upper bound, the nodes with $k_v^+ = 0$ are not driver nodes, as well as the nodes with $k_v^- > 0$ and $k_v^+ = 1$. By neglecting balanced components with all nodes holding $k_v^+ = k_v^- = 1$ with negligible probability, the fraction of driver nodes can be given from the joint degree distribution $P(k_v^- = i, k_v^+ = j) = P_{ij}$, which is

$$\begin{aligned} n_{\rm D}^{\rm DU} &= 1 - \sum_{i=0}^{\infty} P_{i0} - \sum_{i=1}^{\infty} P_{i1} \\ &= 1 - P_0 - P_1 + P_{01}, \end{aligned} \tag{S25}$$

i.e., we remove the joint probabilities for the cases with $k_v^+ = 0$, or with $k_v^- > 0$ and $k_v^+ = 1$ simultaneously. The fraction of driven edges is given by

$$\begin{aligned} m_{\rm D}^{\rm DU} &= \frac{1}{M}\left[M - N\left(1 - \sum_{i=1}^{\infty} P_{i0} - \sum_{j=1}^{\infty} P_{0j} - P_{00}\right)\right] \\ &= 1 - \frac{1}{\langle k \rangle}\left(1 - 2\sum_{i=0}^{\infty} P_{i0} + P_{00}\right) \\ &= 1 - \frac{1}{\langle k \rangle}\left(1 - 2P_0 + P_{00}\right), \end{aligned} \tag{S26}$$

i.e., we remove joint probabilities when in-degree and/or out-degree are/is zero. Here, the average degree is $\langle k \rangle = \langle k^{\rm in} \rangle = \langle k^{\rm out} \rangle = M/N$.

For a directed network with structural switching matrices corresponding to the lower bound, we have proved that our GSBD framework can reproduce the structural edge controllability in directed networks. Thus, the fraction of driver nodes is given by [38]

$$n_{\rm D}^{\rm DL} = \sum_{i=0}^{\infty} \sum_{j=i+1}^{\infty} P_{ij} = \frac{1}{2}\left(1 - \sum_{i=0}^{\infty} P_{ii}\right), \tag{S27}$$

i.e., the fraction of driver nodes is equal to the half of the fraction of non-balanced nodes. The fraction

of driven edges is given by [38]

$$
\begin{aligned}
m_{\mathrm{D}}^{\mathrm{DL}} &= \frac{N}{M}\sum_{i=0}^{\infty}\sum_{j=i+1}^{\infty}(j-i)P_{ij} \\
&= \frac{1}{\langle k\rangle}\sum_{i=0}^{\infty}\sum_{j=1}^{\infty} jP_{i,(i+j)}.
\end{aligned} \tag{S28}
$$

The above equation is derived based on the fact that there are NP_{ij} divergent nodes with in-degree i and out-degree j, and each of them has to drive $j-i$ edges.

For an undirected network with unweighted switching matrices corresponding to the upper bound, components only consisting of the nodes with $k_v^+ = k_v^- = 1$ are not negligible. Note that the number of the components is determined by the number of isolated edges, i.e. the edge does not connect to any other edges in the original undirected network. The fraction of isolated edges can be evaluated by the joint probability $P(i,j)$ in the undirected network, i.e.,

$$
P(1,1) = P_n(1)P_n(1) = \frac{P(1)^2}{\langle k\rangle^2} = \frac{P_1^2}{\langle k\rangle^2}, \tag{S29}
$$

where $P(1,1)$ is the probability of an edge e_{ij} with $k_i = k_j = 1$, $P(k)$ denotes the degree distribution of the undirected network, and $P_n(k)$ denotes the excess degree distribution of the undirected network, that is, the degree distribution for a node at the end of a randomly chosen link. Here, the average degree $\langle k\rangle$ of an undirected network is equal to $\langle k^{\mathrm{in}}\rangle$ and $\langle k^{\mathrm{out}}\rangle$ of the correspondent bidirectional network.

The isolated nodes without edges are not driver nodes, similar to the nodes with $k_v^+ = k_v^- = 1$. Thus the fraction of driver nodes is given by

$$
\begin{aligned}
n_{\mathrm{D}}^{\mathrm{BU}} &= \frac{1}{N}\left(N - NP_0 - NP_1 + \frac{1}{2}MP(1,1)\right) \\
&= 1 - P_0 - P_1 + \frac{1}{2\langle k\rangle}P_1^2,
\end{aligned} \tag{S30}
$$

where we remove the faction of isolated nodes and the nodes with $k_v^+ = k_v^- = 1$ from the drive node set, and include the probability of finding components composed of the nodes with $k_v^+ = k_v^- = 1$. M_{D} of an undirected network with unweighted switching matrices is mainly determined by the number of the nodes with nonzero degree. The fraction of driven edges is given by

$$
\begin{aligned}
m_{\mathrm{D}}^{\mathrm{BU}} &= \frac{1}{M}\left(M - N(1-P_0) + \frac{1}{2}MP(1,1)\right) \\
&= 1 - \frac{1}{\langle k\rangle}(1-P_0) + \frac{1}{2\langle k\rangle^2}P_1^2,
\end{aligned} \tag{S31}
$$

where the probability of the presence of isolated nodes is removed and the probability that components consisting of the nodes with $k_v^+ = k_v^- = 1$ is included.

For an undirected network with structural switching matrices corresponding to the lower bound, the minimum numbers of driver nodes N_{D} and driven edges M_{D} are determined by the number of connected components. For an undirected network with $c = M/N$, the fraction of connected components $n_{\mathrm{CC}} = N_{\mathrm{CC}}/N$ is given by [44]

$$n_{\mathrm{CC}} = \begin{cases} 1-c & \text{if} \quad 0 \leq c \leq \frac{1}{2}, \\ \frac{1}{2c}\left(x(c) - \frac{x(c)^2}{2}\right) & \text{if} \quad c > \frac{1}{2}, \end{cases} \tag{S32}$$

where $x(c) = \sum_{k=1}^{\infty} \frac{k^{k-1}}{k!}(2ce^{-2c})^k$. The fraction of driver nodes is given by

$$n_{\mathrm{D}}^{\mathrm{BL}} = n_{\mathrm{CC}} - P_0, \tag{S33}$$

i.e., we remove the probability for the fact that the connected components are isolated nodes. The fraction of driven edges is given by

$$\begin{aligned} m_{\mathrm{D}}^{\mathrm{BL}} &= \frac{N}{M}(n_{\mathrm{CC}} - P_0) \\ &= \frac{1}{\langle k \rangle}(n_{\mathrm{CC}} - P_0). \end{aligned} \tag{S34}$$

In the following, we combine our general formulas of n_{D} and m_{D} with degree distributions of networks to offer analytical results of three representative networks, including random networks, scale-free networks and networks with an exponential degree distribution.

Erdős-Rényi networks. Directed and undirected Erdős-Rényi (ER) networks are generated by static model [52]. For directed ER networks, both the in- and out-degrees follow a Poisson distribution. Thus, for the upper bound of directed ER network, the expected fraction of driver nodes is given by

$$n_{\mathrm{D}}^{\mathrm{DU}} = 1 - (\langle k \rangle + 1)e^{-\langle k \rangle} + \langle k \rangle \, e^{-2\langle k \rangle}. \tag{S35}$$

The expected fraction of driven edges is

$$m_{\mathrm{D}}^{\mathrm{DU}} = 1 - \frac{1}{\langle k \rangle}(1 - 2e^{-\langle k \rangle} + e^{-2\langle k \rangle}). \tag{S36}$$

For the lower bound of ER directed network, the expected fraction of driver nodes is [38]

$$
\begin{aligned}
n_{\mathrm{D}}^{\mathrm{DL}} &= \frac{1}{2}\left(1-\sum_{i=0}^{\infty}\frac{\langle k\rangle^{2i}}{i!i!}e^{-2\langle k\rangle}\right) \\
&= \frac{1}{2}\left(1-e^{-2\langle k\rangle}I_0(2\langle k\rangle)\right),
\end{aligned}
\tag{S37}
$$

where $I_a(x)$ is the modified Bessel function of the first kind. The expected fraction of driven edges is [38]

$$
\begin{aligned}
m_{\mathrm{D}}^{\mathrm{DL}} &= \frac{e^{-2\langle k\rangle}}{\langle k\rangle}\sum_{i=0}^{\infty}\sum_{j=1}^{\infty} j\frac{\langle k\rangle^{2i+j}}{i!(i+j)!} \\
&= \frac{e^{-2\langle k\rangle}}{\langle k\rangle}\sum_{j=1}^{\infty} jI_j(2\langle k\rangle),
\end{aligned}
\tag{S38}
$$

For undirected ER networks, each undirected edge represents two directed edges with opposite directions. Thus, we have $k_v^+ = k_v^-$ for each node, and both the in- and out-degrees follow the Poisson distribution. For the upper bound of undirected ER networks, the expected fraction of driver nodes is

$$
n_{\mathrm{D}}^{\mathrm{BU}} = 1-(\langle k\rangle+1)e^{-\langle k\rangle}+\frac{\langle k\rangle}{2}e^{-2\langle k\rangle}. \tag{S39}
$$

The expected fraction of driven edges is

$$
m_{\mathrm{D}}^{\mathrm{BU}} = 1-\frac{1}{\langle k\rangle}\left(1-e^{-\langle k\rangle}\right)+\frac{1}{2}e^{-2\langle k\rangle}. \tag{S40}
$$

For the lower bound of undirected ER networks, the expected fraction of driver nodes is

$$
n_{\mathrm{D}}^{\mathrm{BL}} = n_{\mathrm{CC}}-e^{-\langle k\rangle}, \tag{S41}
$$

and the expected fraction of driven edges is

$$
m_{\mathrm{D}}^{\mathrm{BL}} = \frac{1}{\langle k\rangle}\left(n_{\mathrm{CC}}-e^{-\langle k\rangle}\right). \tag{S42}
$$

Scale-free networks based on static model. Directed and undirected scale-free (SF) networks are

generated by static model [52]. Both the in- and out-degrees follow a power-law distribution [53], i.e.,

$$P(k) = \frac{[\langle k\rangle (1-a)]^{1/a}}{a} \frac{\Gamma(k-1/a, \langle k\rangle (1-a))}{\Gamma(k+1)}, \tag{S43}$$

where $\Gamma(z,a)$ is the incomplete Gamma function and $\Gamma(z,a) \to \Gamma(z)$ for $z \to \infty$. We let δ denote $\frac{[\langle k\rangle(1-a)]^{1/a}}{a}$ and let Γ_k denote $\frac{\Gamma(k-1/a,\langle k\rangle(1-a))}{\Gamma(k+1)}$.

For the upper bound of directed SF networks, the expected fraction of driver nodes is

$$n_{\mathrm{D}}^{\mathrm{DU}} = 1 - \delta(\Gamma_0 + \Gamma_1) + \delta^2\Gamma_0\Gamma_1 \tag{S44}$$

The expected fraction of driven edges is

$$m_{\mathrm{D}}^{\mathrm{DU}} = 1 - \frac{1}{\langle k\rangle}\left(1 - 2\delta\Gamma_0 + \delta^2\Gamma_0^2\right). \tag{S45}$$

For the lower bound of directed SF networks, the expected fraction of driver nodes is

$$n_{\mathrm{D}}^{\mathrm{DL}} = \frac{1}{2}\left(1 - \delta^2\sum_{i=0}^{\infty}\Gamma_i^2\right). \tag{S46}$$

The expected fraction of driven edges is

$$m_{\mathrm{D}}^{\mathrm{DL}} = \frac{\delta^2}{\langle k\rangle}\sum_{i=0}^{\infty}\sum_{j=1}^{\infty} j\Gamma_i\Gamma_{i+j}. \tag{S47}$$

For the upper bound of undirected SF networks, the expected fraction of driver nodes is

$$n_{\mathrm{D}}^{\mathrm{BU}} = 1 - \delta(\Gamma_0 + \Gamma_1) + \frac{1}{2\langle k\rangle}\delta^2\Gamma_1^2. \tag{S48}$$

The expected fraction of driven edges is

$$m_{\mathrm{D}}^{\mathrm{BU}} = 1 - \frac{1}{\langle k\rangle}(1 - \delta\Gamma_0) + \frac{1}{2\langle k\rangle^2}\delta^2\Gamma_1^2. \tag{S49}$$

For the lower bound of undirected SF networks, we use the fraction of connected components n_{CC} in undirected ER network to approximate the expected fraction of driver nodes, yielding

$$n_{\mathrm{D}}^{\mathrm{BL}} = n_{\mathrm{CC}} - e^{-\langle k\rangle}. \tag{S50}$$

The expected fraction of driven edges is

$$m_{\mathrm{D}}^{\mathrm{BL}} = \frac{1}{\langle k \rangle}\left(n_{\mathrm{CC}} - e^{-\langle k \rangle}\right). \tag{S51}$$

Exponentially distributed (EX) networks. The EX networks with an exponential degree distribution are generated by configuration model [54]. Both the in- and out-degrees follow the same exponential distribution, which is

$$P(k_v^+ = k) = P(k_v^- = k) = Ce^{-k/\kappa}, \tag{S52}$$

where $C = 1 - e^{-1/\kappa}$ and $\kappa = 1/\log\frac{1+\langle k \rangle}{\langle k \rangle}$.

For the upper bound of directed EX networks, the expected fraction of driver nodes is

$$\begin{aligned} n_{\mathrm{D}}^{\mathrm{DU}} &= 1 - C - Ce^{-1/\kappa} + C^2 e^{-1/\kappa} \\ &= \langle k \rangle \left(\frac{1}{\langle k \rangle + 1} - \frac{1}{(\langle k \rangle + 1)^2} + \frac{1}{(\langle k \rangle + 1)^3}\right). \end{aligned} \tag{S53}$$

The expected fraction of driven edges is

$$\begin{aligned} m_{\mathrm{D}}^{\mathrm{DU}} &= 1 - \frac{1}{\langle k \rangle}(1 - 2C + C^2) \\ &= 1 - \frac{\langle k \rangle}{(\langle k \rangle + 1)^2}. \end{aligned} \tag{S54}$$

For the lower bound of directed EX networks, the expected fraction of driver nodes is [38]

$$\begin{aligned} n_{\mathrm{D}}^{\mathrm{DL}} &= \frac{1}{2}\left(1 - C^2 \sum_{i=0}^{\infty} e^{-2i/\kappa}\right) \\ &= \frac{1}{2}\left(1 - \frac{1 - e^{-1/\kappa}}{1 + e^{-1/\kappa}}\right) \\ &= \frac{\langle k \rangle}{2\langle k \rangle + 1}. \end{aligned} \tag{S55}$$

The expected fraction of driven edges is [38]

$$
\begin{aligned}
m_{\mathrm{D}}^{\mathrm{DL}} &= \frac{C^2}{\langle k\rangle}\sum_{i=0}^{\infty}\sum_{j=1}^{\infty} j e^{-(2i+j)/\kappa} \\
&= \frac{1}{\langle k\rangle}\frac{1-e^{-1/\kappa}}{1+e^{-1/\kappa}}\sum_{j=1}^{\infty} j e^{-j/\kappa} \\
&= \frac{\langle k\rangle + 1}{2\langle k\rangle + 1}.
\end{aligned} \tag{S56}
$$

For the upper bound of undirected EX networks, the expected fraction of driver nodes is

$$
\begin{aligned}
n_{\mathrm{D}}^{\mathrm{BU}} &= 1 - C - Ce^{-1/\kappa} + \frac{1}{2\langle k\rangle}C^2 e^{-2/\kappa} \\
&= 1 - \frac{1}{\langle k\rangle + 1} - \frac{\langle k\rangle}{(\langle k\rangle+1)^2} + \frac{1}{2\langle k\rangle}\left(\frac{1}{\langle k\rangle+1}\right)^2\left(\frac{\langle k\rangle}{\langle k\rangle+1}\right)^2 \\
&= \frac{\langle k\rangle^2}{(\langle k\rangle+1)^2} + \frac{\langle k\rangle}{2(\langle k\rangle+1)^4}.
\end{aligned} \tag{S57}
$$

The expected fraction of driven edges is

$$
\begin{aligned}
m_{\mathrm{D}}^{\mathrm{BU}} &= 1 - \frac{1}{\langle k\rangle}(1-C) + \frac{1}{2\langle k\rangle^2}C^2 e^{-2/\kappa} \\
&= 1 - \frac{1}{\langle k\rangle}\left(\frac{\langle k\rangle}{\langle k\rangle+1}\right) + \frac{1}{2\langle k\rangle^2}\left(\frac{1}{\langle k\rangle+1}\right)^2\left(\frac{\langle k\rangle}{\langle k\rangle+1}\right)^2 \\
&= \frac{\langle k\rangle}{\langle k\rangle+1} + \frac{1}{2(\langle k\rangle+1)^4}.
\end{aligned} \tag{S58}
$$

For the lower bound of undirected EX networks, we use the fraction of isolated components n_{CC} in ER undirected network to estimate the expected fraction of driver nodes, which is

$$
n_{\mathrm{D}}^{\mathrm{BL}} = n_{\mathrm{CC}} - e^{-\langle k\rangle}. \tag{S59}
$$

The expected fraction of driven edges is

$$
m_{\mathrm{D}}^{\mathrm{BL}} = \frac{1}{\langle k\rangle}\left(n_{\mathrm{CC}} - e^{-\langle k\rangle}\right). \tag{S60}
$$

Scale-free networks based on configuration model. The SF networks with power-law degree distributions are generated by configuration model [54]. Both in- and out-degrees follow the same power-law

degree distribution with scaling exponent γ and an exponential cutoff, which is

$$P(k_v^+ = k) = P(k_v^- = k) = Ck^{-\gamma}e^{-k/\kappa}. \tag{S61}$$

The pure power-law distribution has no cutoff ($\kappa \to \infty$), which is

$$P(k_v^+ = k) = P(k_v^- = k) = Ck^{-\gamma}. \tag{S62}$$

For the upper bound of directed SF networks, the expected fraction of driver nodes is

$$\begin{aligned} n_{\mathrm{D}}^{\mathrm{DU}} &= 1 - P_0 - P_1 + P_{01} \\ &= 1 - Ce^{-1/\kappa} \\ &= 1 - \frac{e^{-1/\kappa}}{\mathrm{Li}_\gamma(e^{-1/\kappa})}, \end{aligned} \tag{S63}$$

where $C = 1/\mathrm{Li}_\gamma(e^{-1/\kappa})$ and $\mathrm{Li}_s(z)$ is the polylogarithm function. The polylogarithm $\mathrm{Li}_s(z)$ reduces to the Riemann zeta function $\zeta(s)$ for $z = 1$. Hence, for the upper bound of pure power-law ($\kappa \to \infty$) distributed directed networks, the expected fraction of driver nodes is simplified as

$$n_{\mathrm{D}}^{\mathrm{DU}} = 1 - \frac{1}{\zeta(\gamma)}. \tag{S64}$$

The expected fraction of driven edges is

$$\begin{aligned} m_{\mathrm{D}}^{\mathrm{DU}} &= 1 - \frac{1}{\langle k \rangle}(1 - 2P_0 + P_{00}) \\ &= 1 - \frac{1}{\langle k \rangle} \\ &= 1 - \frac{\mathrm{Li}_\gamma(e^{-1/\kappa})}{\mathrm{Li}_{\gamma-1}(e^{-1/\kappa})}, \end{aligned} \tag{S65}$$

where $\langle k \rangle = C\mathrm{Li}_{\gamma-1}(e^{-1/\kappa})$. When $\kappa \to \infty$, the expected fraction of driven edges is simplified as

$$m_{\mathrm{D}}^{\mathrm{DU}} = 1 - \frac{\zeta(\gamma)}{\zeta(\gamma - 1)}. \tag{S66}$$

For the lower bound of directed SF networks, the expected fraction of driver nodes is [38]

$$\begin{aligned} n_{\mathrm{D}}^{\mathrm{DL}} &= \frac{1}{2}\left(1 - C^2 \sum_{i=0}^{\infty} k^{-2\gamma} e^{-2k/\kappa}\right) \\ &= \frac{1}{2}\left(1 - C^2 \mathrm{Li}_{2\gamma}(e^{-2/\kappa})\right) \\ &= \frac{1}{2} - \frac{\mathrm{Li}_{2\gamma}(e^{-2/\kappa})}{2\mathrm{Li}_{\gamma}(e^{-2/\kappa})^2}. \end{aligned} \tag{S67}$$

When $\kappa \to \infty$, the expected fraction of driver nodes is simplified as

$$n_{\mathrm{D}}^{\mathrm{DL}} = \frac{1}{2} - \frac{\zeta(2\gamma)}{2\zeta(2\gamma)^2}. \tag{S68}$$

The expected fraction of driven edges is [38]

$$\begin{aligned} m_{\mathrm{D}}^{\mathrm{DL}} &= \frac{C^2}{\langle k \rangle} \sum_{i=1}^{\infty} \sum_{j=1}^{\infty} j i^{-\gamma} e^{-i/\kappa} (i+j)^{-\gamma} e^{-(i+j)/\kappa} \\ &= \frac{\sum_{j=1}^{\infty} j e^{-j/\kappa} \sum_{i=1}^{\infty} \frac{e^{-2i/\kappa}}{i^{\gamma}(i+j)^{\gamma}}}{\mathrm{Li}_{\gamma}(e^{-1/\kappa}) \mathrm{Li}_{\gamma-1}(e^{-1/\kappa})}. \end{aligned} \tag{S69}$$

When $\kappa \to \infty$, the expected fraction of driven edges is simplified as

$$m_{\mathrm{D}}^{\mathrm{DL}} = \frac{\sum_{j=1}^{\infty} j \sum_{i=1}^{\infty} i^{-\gamma} (i+j)^{-\gamma}}{\zeta(\gamma)\zeta(\gamma-1)}. \tag{S70}$$

For the upper bound of undirected SF networks, the expected fraction of driver nodes is

$$\begin{aligned} n_{\mathrm{D}}^{\mathrm{BU}} &= 1 - P_0 - C e^{-1/\kappa} + \frac{1}{2\langle k \rangle} C^2 e^{-2/\kappa} \\ &= 1 - \frac{e^{-1/\kappa}}{\mathrm{Li}_{\gamma}(e^{-1/\kappa})} + \frac{e^{-2/\kappa}}{2\mathrm{Li}_{\gamma}(e^{-1/\kappa}) \mathrm{Li}_{\gamma-1}(e^{-1/\kappa})}. \end{aligned} \tag{S71}$$

When $\kappa \to \infty$, the expected fraction of driver nodes is simplified as

$$n_{\mathrm{D}}^{\mathrm{BU}} = 1 - \frac{1}{\zeta(\gamma)} + \frac{1}{2\zeta(\gamma)\zeta(\gamma-1)}. \tag{S72}$$

The expected fraction of driven edges is

$$\begin{aligned} m_{\mathrm{D}}^{\mathrm{BU}} &= 1 - \frac{1}{\langle k \rangle}(1 - P_0) + \frac{1}{2\langle k \rangle^2} C^2 e^{-2/\kappa} \\ &= 1 - \frac{\mathrm{Li}_{\gamma}(e^{-1/\kappa})}{\mathrm{Li}_{\gamma-1}(e^{-1/\kappa})} + \frac{e^{-2/\kappa}}{2\mathrm{Li}_{\gamma-1}(e^{-1/\kappa})^2}. \end{aligned} \tag{S73}$$

When $\kappa \to \infty$, the expected fraction of driven edges is simplified as

$$m_{\mathrm{D}}^{\mathrm{BU}} = 1 - \frac{\zeta(\gamma)}{\zeta(\gamma-1)} + \frac{1}{2\zeta(\gamma-1)^2}. \tag{S74}$$

For the lower bound of power-law and pure power-law ($\kappa \to \infty$) distributed undirected networks, the expected fraction of driver nodes is

$$n_{\mathrm{D}}^{\mathrm{BL}} = n_{\mathrm{CC}} - P_0 = n_{\mathrm{CC}}, \tag{S75}$$

The expected function of driven edges is

$$\begin{aligned} m_{\mathrm{D}}^{\mathrm{BL}} &= \frac{1}{\langle k \rangle}(n_{\mathrm{CC}} - P_0) \\ &= \frac{\mathrm{Li}_{\gamma}(e^{-1/\kappa})}{\mathrm{Li}_{\gamma-1}(e^{-1/\kappa})} n_{\mathrm{CC}}. \end{aligned} \tag{S76}$$

When $\kappa \to \infty$, the expected fraction of driven edges is simplified as

$$m_{\mathrm{D}}^{\mathrm{BL}} = \frac{\zeta(\gamma)}{\zeta(\gamma-1)} n_{\mathrm{CC}}. \tag{S77}$$

Regular networks. The minimum numbers of driver nodes and driven edges in regular networks can be simply calculated in terms of local structural information. For instance, a directed chain graph has $N-2$ nodes with $k_v^+ = k_v^- = 1$, a starting node with $k_v^+ = 1$ and $k_v^- = 0$, and an ending node with $k_v^+ = 0$ and $k_v^- = 1$. Thus, the starting node is a driver node for both upper and lower bounds, and an outgoing edge of this driver node is a driven edge. An undirected chain graph has $N-2$ nodes with $k_v^+ = k_v^- = 2$ and two terminal nodes with $k_v^+ = k_v^- = 1$. Thus, for the upper bound, $N-2$ nodes are driver nodes and each driver node has to drive one outgoing edge; for the lower bound, the undirected graph is a balanced component and only one driver node and one driven edge are required. The simple analytical results of several regular networks are shown in Tab. S1.

Supplementary Note 6: Transition between the upper and lower bounds

We explore the transition between the upper and the lower bound. Without loss of generality and for simplicity, we only consider a single switching matrix S_v and adjust the element values in S_v to realize the transition from the upper bound to the lower bound of $\mathrm{rank}(S_v)$. If the upper (lower) bounds of $\mathrm{rank}(S_v)$ for all switching matrices are reached, the lower (upper) bounds of n_D and m_D for the whole network are achieved as well.

The lower bound of $\mathrm{rank}(S_v)$ can be reached by assigning elements with identical values in the switching matrix S_v with dimension $L \times \mu L$ where $\mu \geqslant 1$. We study the normalized rank $(1/L)\mathrm{rank}(S_v)$ as a function of the proportion $\rho \in [0, 1]$ of random values in S_v. In other words, ρ is the fraction of elements with random values in S_v and there are only elements with identical values or random values in S_v.

To study the transition between the upper and lower bounds of $(1/L)\mathrm{rank}(S_v)$, a formula in terms of balls associated with bins is necessary [55], which is

$$E[z] = (1 - \frac{1}{\beta})^{\alpha}, \tag{S78}$$

where $E[z]$ is the expectation of the number of empty bins when α balls are placed randomly into β bins.

We assume that each row of S_v is an empty bin, and the emergence of an element with random value is associated with a ball falling into an empty bin. Thus, we can use the number of empty bins to approximate the number of linearly dependent rows, and the normalized rank is given by:

$$\frac{1}{L}\mathrm{rank}(S_v) = 1 - \left(1 - \frac{1}{L}\right)^{\rho\mu L^2}, \tag{S79}$$

As shown in Fig. S4(a) and (b), the analytical prediction from the above equation is in reasonable agreement with numerical results for switching matrices with different dimensions.

Both the simulation and analytical results demonstrate that the velocity of the transition from the lower bound to the upper bound as ρ increases is determined by the higher value between the column dimension and the row dimension, or in other words, determined by the larger value between in- and out-degree of a node that is associated with the dimensions of the switching matrix. In general, a node with a larger incoming or outgoing degree is associated with a fast transition from the lower to the upper bound through increasing the fraction of the elements with random values, as shown in Fig. S4.

Supplementary Note 7: Strong structural controllability

In an arbitrary network, a node is said to be strongly structurally controllable (SSC) if, whatever values the elements in its switching matrix take, the category of the node and that of its outgoing edges will not change [19, 40]. Similarly, a node is said to be weakly structurally controllable (WSC) if there exist a switching matrix of this node is not of full rank for a set of elements values. The numbers of strongly and weakly structurally controllable nodes are denoted by N_{SSC} and N_{WSC}, respectively. We formulate a theorem as follows.

Theorem 3 *In GSBD, the strongly structurally controllable nodes include the nodes with $k_v^+ \leq 1$ or $k_v^- \leq 1$, and the weakly structurally controllable nodes include the nodes with $k_v^+ > 1$ and $k_v^- > 1$.*

Proof. In the GSBD, switching matrix does not exist for a node with $k_v^+ = 0$ or $k_v^- = 0$, so that the change of nonzero elements has no influence to the category of the node and of its outgoing edges. For a node with $k_v^+ > 0$ and $k_v^- > 0$, the minimum rank of its switching matrix is one. The change of nonzero elements will not affect the category of the nodes with $k_v^+ = 1$ or $k_v^- = 1$ and that of its outgoing edges. In contrast, the rank of switching matrix can change for a node with $k_v^+ > 1$ and $k_v^- > 1$. These conclude our proof: for an arbitrary network, the strongly structurally controllable nodes include the nodes with $k_v^+ \leq 1$ or $k_v^- \leq 1$, and the weakly structurally controllable nodes include the nodes with $k_v^+ > 1$ and $k_v^- > 1$, and $N = N_{\mathrm{SSC}} + N_{\mathrm{WSC}}$.

Analytical results of N_{ssc}. The dependence of the N_{ssc} on the joint degree distribution allows us to derive analytical formulas for the expected fraction of SSC nodes in model networks. The fraction of SSC nodes in a directed network with a joint degree distribution $P(k_v^- = i, k_v^+ = j) = P_{ij}$ can be formulated as

$$\begin{aligned} n_{\mathrm{SSC}} &= \sum_{i=0}^{\infty} P_{i0} + \sum_{i=0}^{\infty} P_{0j} - P_{00} + \sum_{i=1}^{\infty} P_{i1} + \sum_{i=1}^{\infty} P_{1j} - P_{11} \\ &= 2\left(\sum_{i=0}^{\infty} P_{i0} + \sum_{i=1}^{\infty} P_{i1}\right) - P_{00} - P_{11} \\ &= 2(P_0 + P_1 - P_{01}) - P_{00} - P_{11}, \end{aligned} \tag{S80}$$

where we include the joint probabilities for the case associated with $k_v^+ \leq 1$ or $k_v^- \leq 1$, and remove the repeating parts. Then the fraction of WSC nodes is given by $n_{\mathrm{WSC}} = 1 - n_{\mathrm{SSC}}$.

The expected fraction of SSC nodes in an undirected network is

$$n_{\text{SSC}} = P_0 + P_1. \tag{S81}$$

where we include the probabilities of the case associated $k_v = 0$ and $k_v = 1$. Then we obtain the expected fraction of WSC nodes via $n_{\text{WSC}} = 1 - n_{\text{SSC}}$.

For directed ER networks, both the in- and out-degrees follow a Poisson distribution. By combining the degree distribution with the general formulas of n_{ssc}, we obtain the expected fraction of SSC nodes, as follows.

$$\begin{aligned} n_{\text{SSC}} &= 2\left(e^{-\langle k\rangle} + \langle k\rangle\, e^{-\langle k\rangle} - \langle k\rangle\, e^{-2\langle k\rangle}\right) - e^{-2\langle k\rangle} - \langle k\rangle^2\, e^{-2\langle k\rangle} \\ &= 2(\langle k\rangle + 1)e^{-\langle k\rangle} - (\langle k\rangle + 1)^2 e^{-2\langle k\rangle}. \end{aligned} \tag{S82}$$

For undirected ER networks, the expected fraction of SSC nodes is given by

$$n_{\text{SSC}} = (\langle k\rangle + 1)e^{-\langle k\rangle}. \tag{S83}$$

For directed SF networks generated by static model [52], both the in- and out-degrees follow a power-law distribution. By combining the degree distribution with the general formulas of n_{ssc} and assuming the absence of the correlation between in- and out-degrees of nodes, we obtain the expected fraction of SSC nodes, as follows.

$$\begin{aligned} n_{\text{SSC}} &= 2(\delta\Gamma_0 + \delta\Gamma_1 - \delta^2\Gamma_0\Gamma_1) - \delta^2(\Gamma_0^2 + \Gamma_1^2) \\ &= 2\delta(\Gamma_0 + \Gamma_1) - \delta^2(2\Gamma_0\Gamma_1 + \Gamma_0^2 + \Gamma_1^2). \end{aligned} \tag{S84}$$

For undirected SF networks, the expected fraction of SSC nodes is

$$n_{\text{SSC}} = \delta(\Gamma_0 + \Gamma_1). \tag{S85}$$

For directed exponentially distributed networks, both the in- and out-degrees follow the same exponential distribution. The expected fraction of SSC nodes is

$$\begin{aligned} n_{\text{SSC}} &= 2\left(C + Ce^{-1/\kappa} - C^2 e^{-1/\kappa}\right) - C^2 - C^2 e^{-2/\kappa} \\ &= \frac{4\langle k\rangle + 1}{(\langle k\rangle + 1)^2} - \frac{3\langle k\rangle^2 + 2\langle k\rangle}{(\langle k\rangle + 1)^4}. \end{aligned} \tag{S86}$$

For undirected exponentially distributed networks, the expected fraction of SSC nodes is given by

$$\begin{aligned} n_{\mathrm{SSC}} &= C + Ce^{-1/\kappa} \\ &= \frac{2\langle k\rangle + 1}{(\langle k\rangle + 1)^2}. \end{aligned} \tag{S87}$$

For the directed SF networks generated by configuration model [54], both the in- and out-degrees follow a power-law degree distribution with a scaling exponent γ and an exponential cutoff. The expected fraction of SSC nodes for finite κ is

$$\begin{aligned} n_{\mathrm{SSC}} &= 2Ce^{-1/\kappa} - C^2 e^{-2/\kappa} \\ &= 2\frac{e^{-1/\kappa}}{\mathrm{Li}_\gamma(e^{-1/\kappa})} - \frac{e^{-2/\kappa}}{\mathrm{Li}_\gamma(e^{-1/\kappa})^2}. \end{aligned} \tag{S88}$$

When $\kappa \to \infty$, the expected fraction of SSC nodes is simplified as

$$n_{\mathrm{SSC}} = 2\frac{1}{\zeta(\gamma)} - \frac{1}{\zeta(\gamma)^2}. \tag{S89}$$

For the undirected SF networks generated by configuration model, the expected fraction of SSC nodes for finite κ reads

$$n_{\mathrm{SSC}} = Ce^{-1/\kappa} = \frac{e^{-1/\kappa}}{\mathrm{Li}_\gamma(e^{-1/\kappa})}. \tag{S90}$$

When $\kappa \to \infty$, the expected fraction of SSC nodes is simplified as

$$n_{\mathrm{SSC}} = \frac{1}{\zeta(\gamma)}. \tag{S91}$$

For regular networks, N_{ssc} can be exactly calculated based on local information. For instance, a directed chain graph has $N-2$ nodes with $k_v^+ = k_v^- = 1$, a starting node with $k_v^+ = 1$ and $k_v^- = 0$, and an ending node with $k_v^+ = 0$ and $k_v^- = 1$. Thus all nodes are SSC in the directed chain graph. An undirected chain graph has $N-2$ nodes with $k_v^+ = k_v^- = 2$ and two terminal nodes with $k_v^+ = k_v^- = 1$. According to the criterion of SSC nodes, there are two SSC nodes corresponding to the two terminal nodes. The analytical results of the other regular networks are shown in Tab. S1.

Supplementary Note 8: Theoretical predictions of real networks

We substantiate how to derive theoretical predictions of driver nodes n_{D}, driven edges m_{D} and strong structural controllability n_{ssc} for real directed and undirected networks. To be specific, we insert the in- and out-degree distribution of a real directed network into Eq. (S25) to predict the fraction of driver nodes $n_{\mathrm{D}}^{\mathrm{analyse}}$ in the upper bound via

$$\begin{aligned} n_{\mathrm{D}}^{\mathrm{analyse}} &= 1 - \sum_{i=0}^{\infty} P_{i0} - \sum_{i=1}^{\infty} P_{i1} \\ &= 1 - P_{\mathrm{out}}(0) - P_{\mathrm{out}}(1) + P_{\mathrm{in}}(0) P_{\mathrm{out}}(1), \end{aligned} \tag{S92}$$

where $P_{\mathrm{out}}(0)$, $P_{\mathrm{out}}(1)$ and $P_{\mathrm{in}}(0)$ are the fraction of nodes in the real network with degree $k_v^+ = 0$, $k_v^+ = 1$ and $k_v^- = 0$, respectively. The theoretical predictions of the fraction of driven edges $m_{\mathrm{D}}^{\mathrm{analyse}}$ and strong structural controllability $n_{\mathrm{scc}}^{\mathrm{analyse}}$ are given in a similar way.

Supplementary Note 9: Network models and data sets

Static model. Directed and undirected ER and SF networks can be generated by static model [52].

A directed ER network starts from N isolated nodes, and the identical weight $1/N$ was assigned to each node, leading to the same selected probability of each node. We randomly select two nodes and connect them with a directed edge e_{ij} from node i to j if there exists no edge between them. This process is repeated until $|E| = M$ edges are created in the network. The ER networks generated in this way exhibits the Poisson distribution of both in-degrees and out-degrees, as follows:

$$P(k) = \frac{e^{-\langle k \rangle} \langle k \rangle^k}{k!}, \tag{S93}$$

where the average degree is $\langle k \rangle = \langle k^+ \rangle = \langle k^- \rangle = M/N$.

A directed SF network starts from N isolated nodes. We assign two weights $p_i = i^{-a_{\text{out}}}$ and $q_i = i^{-a_{\text{in}}}$ $(i = 1, ..., N)$ to each node, respectively, where a_{in}, $a_{\text{out}} \in (0, 1)$. Then two nodes i and j, are selected with probabilities $p_i / \sum_k p_k$ and $q_j / \sum_k q_k$ $(k = 1, ..., N)$, respectively, and they are connected by a directed edge e_{ij} from node i to j if there exists no edge between them. This process is repeated until $|E| = M$ edges are created in the network. The in-degree or out-degree distributions of the SF network are [53]

$$P(k) = \frac{[\langle k \rangle (1-a)]^{1/a}}{a} \frac{\Gamma(k - 1/a, \langle k \rangle (1-a))}{\Gamma(k+1)}, \tag{S94}$$

where $\Gamma(s, x)$ is the incomplete Gamma function, $\Gamma(n) = (n-1)!$ is the gamma function, and $a = a_{\text{in}} = a_{\text{out}}$. For large k, the above formula gives the asymptotic behavior of the degree distribution, which is

$$P(k) \simeq \frac{[\langle k \rangle (1-a)]^{1/a}}{a} \frac{\Gamma(k - 1/a)}{\Gamma(k+1)} \sim k^{-1-1/a}. \tag{S95}$$

The SF network generated in this way follows a power law distribution of both the in-degrees and out-degrees, and the scaling exponents are $\gamma^{\text{in}} = (1 + a_{\text{in}})/a_{\text{in}}$ and $\gamma^{\text{out}} = (1 + a_{\text{out}})/a_{\text{out}}$, respectively.

Note that, for a SF network generated by static model, its in-degree and out-degree of a node are correlated, i.e., a node with a large in-degree usually has a large out-degree (the first node $i = 1$ has the maximum in-degree and out-degree). To eliminate the correlation, after assigning two weights $i^{-a_{in}}$ and $i^{-a_{out}}$ to each node, we randomly reset the order of both weight sequences. As a result, after the reset, the weights of first node may be $p_1 = i^{-a_{out}}$ and $q_1 = j^{-a_{in}}$, where $i \neq j \neq 1$ and $0 < i, j \leq N$. Thus, the correlation between in- and out-degrees of any nodes will be negligible in the SF network.

The ER and SF undirected networks are generated in the same way as the directed networks.

Configuration model. EX and SF networks can be generated by configuration model [54].

For an undirected network with a given expected degree sequence $K = (k_1, k_2, ..., k_n)$ following an exponential or power-law distribution, every node v_i is assigned weight k_i. The probability of establishing an edge e_{ij} is according to the joint weights of the nodes at both ends:

$$P_{ij} = \frac{k_i k_j}{\sum_l k_l}, \tag{S96}$$

where $\max(k_i^2) < \sum_l k_l$, and the k_i is not limited to be integers.

The directed EX and SF networks have a similar generating process as above. Two expected degree sequences k^+ and k^- following an exponential or power-law distribution are given, and a node v_i is assigned node weight k_i^+ and k_i^-. We reset the order of both weight sequences stochastically to eliminate the correlation between in- and out-degrees of each node in directed networks. The probability of creating an edge e_{ij} is according to the joint weights of the nodes at both ends:

$$P_{ij} = \frac{k_i^+ k_j^-}{\sum_l k_l}, \tag{S97}$$

where $\max(k_i^+ k_j^-) < \sum_l k_l$, and k_i^+ and k_i^- are not limited to be integers.

Real networks. The details of the real-world directed and undirected networks in the main text and Supplementary Information are presented in Tab. S2. For each real network, we show its type, name, the number of nodes, the number of edges, and physical description. Note that, to be sufficiently clear, we give the semantics of edges in directed networks.

Supplementary References

46. Rugh, W. J. in *Linear System theory*, 2nd edn (Prentice-Hall, 1996).

47. Antsaklis, P. J. & Michel, A. N. *Linear Systems* (McGraw-Hill, 1997).

48. Hautus, M. L. J. Controllability and observability conditions of linear autonomous systems. *Ned. Akad. Wetenschappen, Proc. Ser. A* **72**, 443–448 (1969).

49. Papoulis, A. & Pillai, S. U. *Probability, Random Variables and Stochastic Processes* (McGraw-Hill, 2002).

50. Strang, G. *Linear Algebra and Its Applications* (Thomson Brooks/Cole, 2006).

51. Bang-Jensen, J. & Gutin, G. Z. *Digraphs: theory, algorithms and applications* (Springer, 2008).

52. Goh, K. I., Kahng, B. & Kim, D. Universal behavior of load distribution in scale-free networks. *Phys. Rev. Lett.* **87**, 278701 (2001).

53. Catanzaro, M. & Pastor-Satorras, R. Analytic solution of a static scale-free network model. *Eur. Phys. J. B* **44**, 241–248 (2005).

54. Chung, F. & Lu, L. Connected components in random graphs with given expected degree sequences. *Ann. Combin.* **6**, 125–145 (2002).

55. Kamath, A., Motwani, R., Palem, K. & Spirakis, P. Tail bounds for occupancy and the satisfiability threshold conjecture. *Random. Struct. & Algor.* **7**, 59–80 (1995).

56. Milo, R., Itzkovitz, S., Kashtan, N., Levitt, R., Shen-Orr, S., Ayzenshtat, I., Sheffer, M. & Sheffer, M. Superfamilies of evoved and designed networks. *Science* **303**, 1538–1542 (2004).

57. Van Duijn, M. A. J., Zeggelink, E. P. H., Huisman, M., Stokman, F. N. & Wasseur, F. W. Evolution of sociology freshmen into a friendship network. *J. Math. Sociol.* **27**, 153–191 (2003).

58. Baird, D., Luczkovich, J. & Christian, R. R. Assessment of spatial and temporal variability in ecosystem attributes of the St Marks National Wildlife Refuge, Apalachee Bay. *Florida. Estuarine, Coastal, and Shelf Science* **47**, 329–349 (1998).

59. Christian, R. R. & Luczkovich, J. J. Organizing and understanding a winters seagrass food web network through effective trophic levels. *Ecol. Model.* **117**, 99–124 (1999).

60. Dunne, J. A., Williams, R. J. & Martinez, N. D. Food-web structure and network theory: the role of connectance and size. *Proc. Natl. Acad. Sci. USA* **99**, 12917–12922 (2002).

61. http://pil.phys.uniroma1.it/∼ gcalda/cosinsite/extra/data/food-webs/WEB.html.

62. Martinez, N. D. Artifacts or attributes? Effects of resolution on the Little Rock Lake food web. *Ecol. Monogr.* **61**, 367–392 (1991).

63. Watts, D. J. & Strogatz, S. H. Collective dynamics of small-world networks. *Nature* **393**, 440-442 (1998).

64. http://vlado.fmf.uni-lj.si/pub/networks/data/cite/default.htm.

65. http://www.cise.ufl.edu/research/sparse/matrices/Pajek/Kohonen.html.

66. Adamic, L. A. & Glance, N. The Political Blogosphere and the 2004 US Election: divided they blog. *Proceedings of the 3rd international workshop on Link discovery*, 36–43 (2005).

67. Leskovec, J., Kleinberg, J. & Faloutsos, C. Graph evolution: Densification and shrinking diameters. *ACM Transactions on Knowledge Discovery from Data(TKDD)* **1**, 2 (2007).

68. Ripeanu, M., Foster, I. & Iamnitchi, A. Mapping the gnutella network: Properties of large-scale peer-to-peer systems and implications for system design. *arXiv preprint*, cs/0209028 (2002).

69. Freeman, S. C. & Freeman, L. C. The networkers network: A study of the impact of a new communications medium on sociometric structure. *Social Science Research Reports* (1979).

70. Cross, R. L. & Parker, A. The hidden power of social networks: Understanding how work really gets done in organizations. *Harvard Business Review Press* (2004).

71. http://www.clairlib.org/index.php/Corpora.

72. http://vlado.fmf.uni-lj.si/pub/networks/data/mix/USAir97.net.

73. Colizza, V., Pastor-Satorras, R. & Vespignani, A. Reaction-Diffusion processes and meta-population models in Heterogeneous Networks. *Nat. Phys.* **3**, 276–282 (2007).

74. Leskovec, J. & Mcauley, J. J. Learning to discover social circles in ego networks. in *Advances in neural information processing systems*, 539–547 (2012).

75. http://pil.phys.uniroma1.it/gcalda/cosinsite/extra/data/internet/nlanr.html.
